# A protocol for high-quality sectioning for tree-ring anatomy

**DOI:** 10.3389/fpls.2025.1505389

**Published:** 2025-02-26

**Authors:** Marina V. Fonti, Georg von Arx, Maryline Harroue, Loïc Schneider, Daniel Nievergelt, Jesper Björklund, Rashit Hantemirov, Vladimir Kukarskih, Cyrille B.K. Rathgeber, Nadja-Tamara Studer, Patrick Fonti

**Affiliations:** ^1^ Forest Dynamics, Swiss Federal Institute for Forest, Snow and Landscape Research WSL, Birmensdorf, Switzerland; ^2^ Oeschger Centre for Climate Change Research, University of Bern, Bern, Switzerland; ^3^ Université de Lorraine, AgroParisTech, INRAE, SILVA, Nancy, France; ^4^ Institute of Plant and Animal Ecology, Ural Division of the Russian Academy of Sciences, Ekaterinburg, Russia; ^5^ Ural Institute for Humanities, Ural Federal University, Ekaterinburg, Russia

**Keywords:** dendrochronology, quantitative wood anatomy (QWA), subfossil wood, embedding, dendroanatomy, tree-ring anatomy, wood thin sectioning, xylem

## Abstract

Quantitative wood anatomy (QWA), which involves measuring wood cell anatomical characteristics commonly on dated tree rings, is becoming increasingly important within plant sciences and ecology. This approach is particularly valuable for studies that require processing a large number of samples, such as those aimed at millennial-long climatic reconstructions. However, the field faces significant challenges, including the absence of a publicly available comprehensive protocol for efficiently and uniformly producing high-quality wood thin sections for QWA along dated tree-ring series. This issue is especially critical for more brittle subfossil wood, in addition to fresh material from living trees. Our manuscript addresses these challenges by providing a detailed protocol for producing thin anatomical sections of wood and digital images, specifically tailored for long chronologies of tree-ring anatomy with an emphasis on conifer wood. The protocol includes step-by-step procedures for sample preparation, sectioning, and imaging, ensuring consistent and high-quality results. By offering this well-tried-and-tested protocol, we aim to facilitate reproducibility and accuracy in wood anatomical studies, ultimately advancing research in this field. It aims to serve as a reference for researchers and laboratories engaged in similar work, promoting standardized practices and enhancing the reliability of QWA data.

## 1 Introduction

Quantitative measurements of the anatomical features of wood cells (QWA) commonly obtained from dated tree rings have been shown to provide stronger links with inter-annual, as well as intra-seasonal climatic variability than traditional proxies such as ring width (Carrer et al., 2015; Pritzkow et al., 2016; Ziaco et al., 2016; Balanzategui et al., 2021; Allen et al., 2022; Lopez-Saez et al., 2023). By providing data on individual cells constituting each tree ring, this emerging array of proxies has the advantage of offering high-temporal resolution and a more direct mechanistic link between climate, wood structure, and wood function (Hacke et al., 2001; Sperry et al., 2006; Hacke et al., 2015; Rosner et al., 2016a, Rosner et al., 2016b; Castagneri et al., 2017; Prendin et al., 2018), making it particularly suitable for a wide range of applications. These include ecological studies (Puchi et al., 2020; Butto et al., 2021; Olano et al., 2022; Garcia-Gonzalez and Souto-Herrero, 2023), investigations into species and provenance adaptation (Eilmann et al., 2014; Pampuch et al., 2021), for intra-annual dating of specific events such as volcanoes (Edwards et al., 2021; Buntgen et al., 2022; Bessanova et al. 2024) or flooding (Nolin et al., 2021; Tardif et al., 2021; Thaxton et al., 2023), to quantify physiological responses (Sviderskaya et al., 2021; Peters, 2023), and intra-annual tree biomass increase (Martinez-Sancho et al., 2022; Puchi et al., 2023).

The ability of the wooden cells to reflect seasonal climatic conditions is likely due to the strong control of environmental factors on cambial activity (Rathgeber et al., 2016; Carrer et al., 2017; Castagneri et al., 2017; Deslauriers et al., 2017), as well as the possibility of statistically integrating anatomical traits from a population of relatively numerous tiny cells growing at the same time, i.e. cells belonging to a tangential band that can be as narrow as a few tens of micrometers (von Arx et al., 2016; Bjorklund et al., 2020). This improved signal quality is particularly critical for refining palaeoecological reconstructions, as recently shown with a millennial-long chronology of Scots pine tracheid anatomy from Northern Fennoscandia, which demonstrates that current warming far exceeded the medieval warm period (Björklund et al., 2023).

However, achieving such (multi)-millennial-long cell anatomical chronologies requires extensive resources since analyzing and producing data for millions of cells comes at the cost of more complex preparation and tedious measurements. Even though a stronger climatic common signal (that is, similarity among trees in cell responses to the environment) considerably reduces the sample depth necessary to meet the statistical requirements for reconstruction (Wigley et al., 1984), substantial processing of the wood is still needed to obtain specimens suitable for sectioning. Recent and ongoing work like the Fennonscandian QWA dataset (Björklund et al., 2023) comprises 20-30 trees per year between 850-2019 CE (1015 thin sections from 188 trees) and the still unpublished 7.5 millennia-long QWA from the Yamal Peninsula in Siberia (Hantemirov et al., 2022), features an annual sample depth of about 12 trees (3200 thin sections from ~ 650 trees). Furthermore, quality requirements of the samples for QWA are more exigent than for ring-width due to, e.g., the fragility and degradation of subfossil material, and constitute additional challenges to meet replication demands when sample availability decreases back in time (Pearl et al., 2020). A rigorous processing protocol is thus key to ensuring both efficient and consistent high-quality processing of wood samples (von Arx et al., 2016).

The use of QWA for climatic reconstruction but also to support other research questions based on tree-ring series in plant science is gaining popularity among laboratories and among scientists looking to exploit this promising approach (von Arx et al., 2021). This positive trend is also accompanied by increased accessibility to tools (e.g Gartner and Nievergelt, 2010; Arzac et al., 2018), software (e.g (von Arx and Carrer, 2014; Dyachuk et al., 2020), and novel digital measuring approaches [e.g., artificial intelligence (Resente et al., 2021; Katzenmaier et al., 2023)], which are increasingly facilitating wood processing and enabling higher throughput.

Numerous lab and video-supported protocols explaining how to handle samples for the preparation of wood thin sections have already been made available^1^ ^,^ ^2^ ^,^ ^3^ ^,^ ^4^ ^,^ ^5^. For instance (Gärtner and Schweingruber, 2013; Gartner et al., 2015; von Arx et al., 2015), have proposed an excellent overview of different techniques, while (Prislan et al., 2022) recently updated the protocol for sample preparation for wood and phloem formation analyses. Regarding quantitative wood measurements von Arx et al. (2016), presented comprehensive guidelines covering all steps and potential pitfalls along the entire production chain, from sample collection to data analysys, and Reining et al. (2018) introduced anatomical techniques specific to handling ring width measurements in deformed subfossil wood. Recently Frigo et al. (2024), compared embedding versus not embedding techniques using fresh material. Although standardizing technical procedures and homogenizing data production is broadly useful, a specific lab protocol for efficiently producing massive quantities of high-quality, homogeneous thin sections for large datasets (e.g., long chronologies) of tree-ring anatomy is still lacking. Such protocol is needed in most studies typically including heterogeneous wood, and especially for fragile subfossil wood.

**Figure 1:**
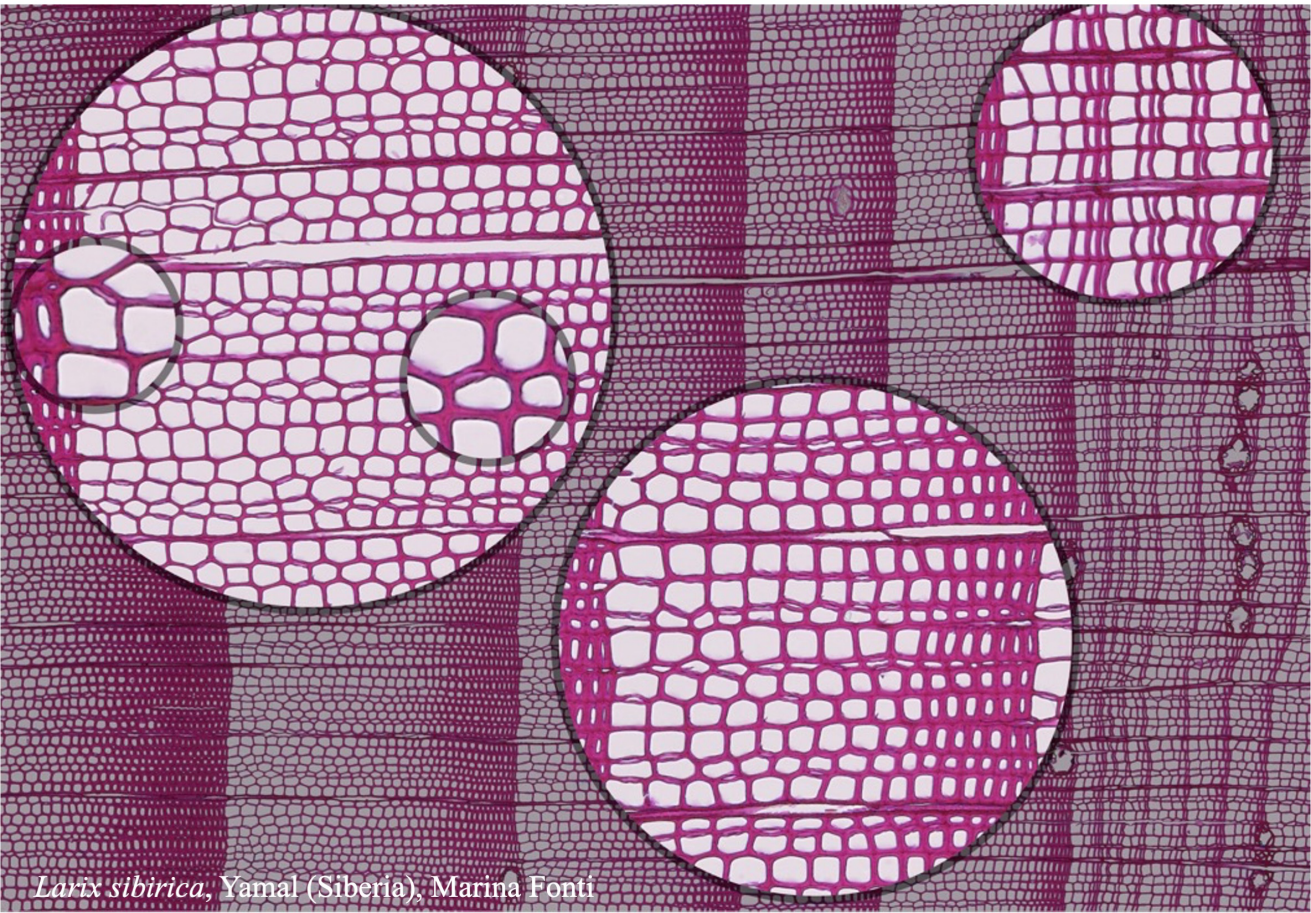
High-quality thin section from subfossil Larix sibirica wood from the Yamal Region in Siberia (Russia). The youngest ring, at the right, dates to 4404 BCE. Inset circles magnify the structural details of rings with varying widths. The field of view (rectangular shaded background image) is 2.381 mm high by 3.440 mm wide. Left to right corresponds to pith-to-bark orientation.

Here, we share the protocol we developed for the mass production of wooden thin sections for millennial-long climatic reconstructions, with an emphasis on conifer wood. This protocol is equally useful for any research application involving extended time series of wood anatomical traits. Initially developed at the INRAE Research Center Grand Est – Nancy for sectioning thin anatomical sections of wood microcores for xylogenesis monitoring (Harroué et al., 2011), this protocol for paraffin-embedded samples has been further adapted at WSL for the production of long chronologies of wood anatomy measurements (an example for such a section is shown in **Figure 1**). While the protocol is particularly suitable for conifer wood, due to the fragility of tracheid cell walls that often require embedding to prevent disruption during sectioning, it is also applicable to angiosperm wood with minor adjustments. These adjustments may account for the greater hardness of hardwoods or specific targeted anatomical features, such as vessels and fiber lumina or cell wall dimensions, which benefit from thinner, high-quality sections. The protocol provides comprehensive instructions for all laboratory processes, including a troubleshooting guide, a suggested weekly processing schedule, and a list of required tools, devices, and materials. By focusing on conifers as a practical example, we aim to emphasize the protocol’s reliability and adaptability for a wide range of wood types and research applications involving extended time series of wood anatomical traits.

## 2 Overview of the full procedure

**Figure 2:**
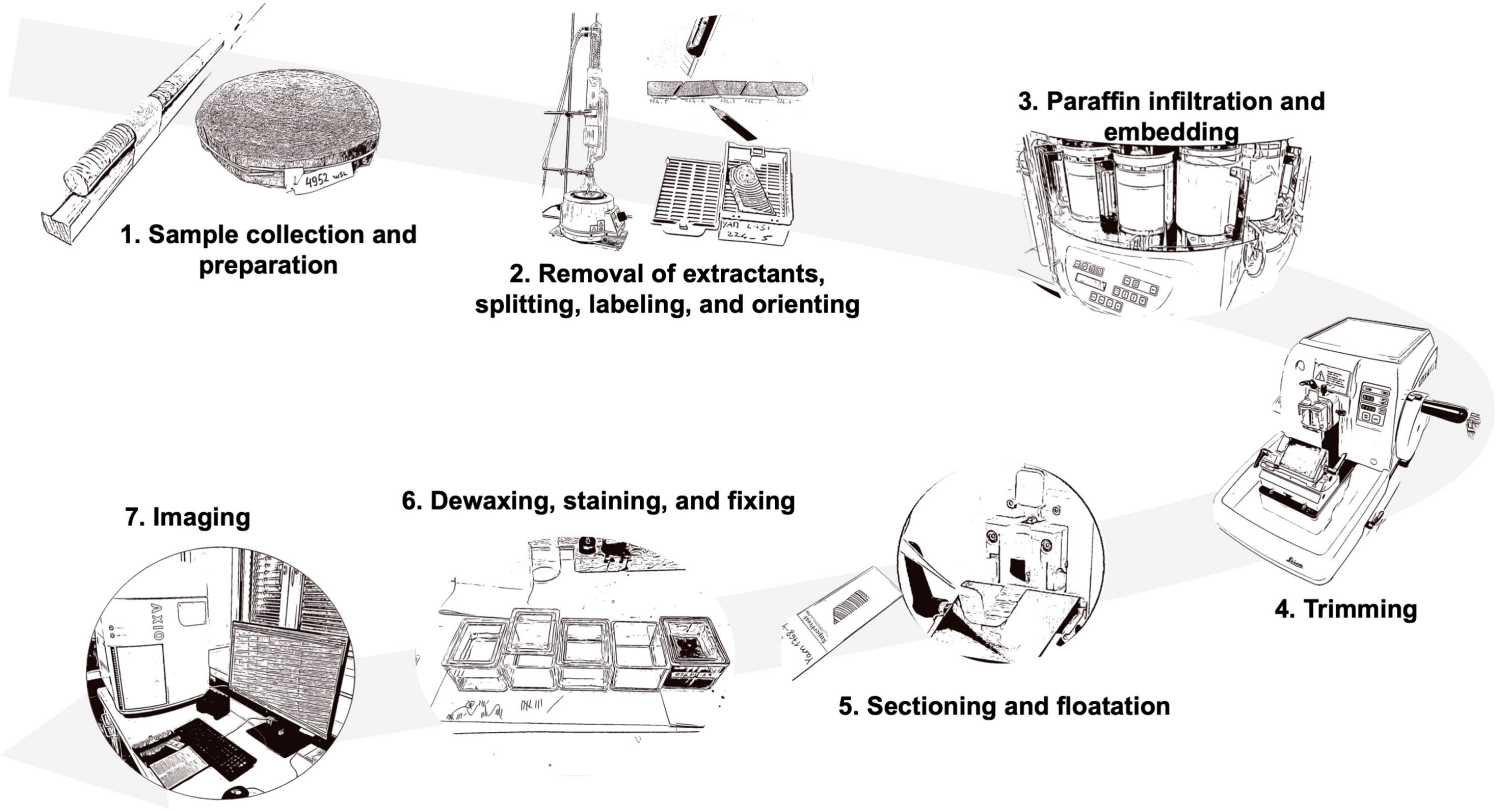
Overview of the full procedure for preparing thin sections of wood for cell anatomical measurements along time-series of tree rings for high-throughput production of high-quality wood histologic thin sections. The protocol includes the following steps: 1. Sample collection and preparation; 2. Removal of extractants, splitting, labeling, and orienting; 3. Paraffin infiltration and embedding; 4. Trimming; 5. Sectioning and floatation; 6. Dewaxing, staining, and fixing; and 7. Imaging.

The protocol focuses on the preparation of thin sections of conifer wood covering: 1. Sample collection and preparation; 2. Removal of extractants, splitting, labeling, and orienting; 3. Paraffin infiltration and embedding; 4. Trimming; 5. Sectioning and floatation; 6 Dewaxing, staining, and fixing; and 7. Imaging (**Figure 2**). Although these steps are commonly used in wood anatomy research, they are often applied to qualitative studies with limited samples. Our protocol, however, is specifically optimized for high-throughput production and for dealing with brittle, heterogeneous materials like subfossil wood. The goal is to produce high-quality histological sections suitable for wood anatomy measurements via image processing techniques.

This protocol emphasizes using high-resolution imaging (e.g., Zeiss Axio Scan.Z1 slide scanner, Carl Zeiss, Germany) to enable semi-automated or AI-based anatomical measurements within dated tree rings [e.g., using ROXAS software (von Arx and Carrer, 2014)]. These images can be further analyzed using open-source tools like R (R Core Team, 2021), facilitating data homogenization and archiving in shared databases.

The preparation process involves consecutive steps requiring skill, experience, and strict adherence to safety measures. Understanding the entire workflow is essential for troubleshooting potential challenges during later stages of the process. This step-by-step protocol was developed as a comprehensive reference guide based on the methods used in our own wood anatomy laboratories and includes common issues and solutions. It accommodates the specificities of fresh, dry, or subfossil wood samples collected as radial cores or wooden discs.

To ensure a safe working environment while conducting these laboratory procedures, always adhere to the Biosafety Level 1 (BSL-1) guidelines (http://www.cdc.gov/labs/bmbl) and refer to the user manuals and guidelines of the specific instrument you are using (**Supplementary Table 1** for those used in this protocol). This will help minimize risks to personnel and the environment.

### 2.1 Sample collection and preparation

To prepare thin sections, the collected wood samples must be cleaned of resin and other extractants, and then split into pieces small enough to accommodate the subsequent processing steps. Before splitting, we recommend measuring the tree-ring widths for easier subsequent dating verification of the anatomical cross-sections. Proper preparation involves three key aspects: ensuring that the pieces are of a size suitable for handling and compatible with the rotary microtome, properly aligning the wood samples to maintain consistent orientation throughout the process, and clearly labeling each piece to facilitate easy identification and re-orientation during subsequent steps. This ordered approach accounts for the limitation of maximum sample dimensions and re-orientation constraints associated with using a rotary microtome.

**Figure 3:**
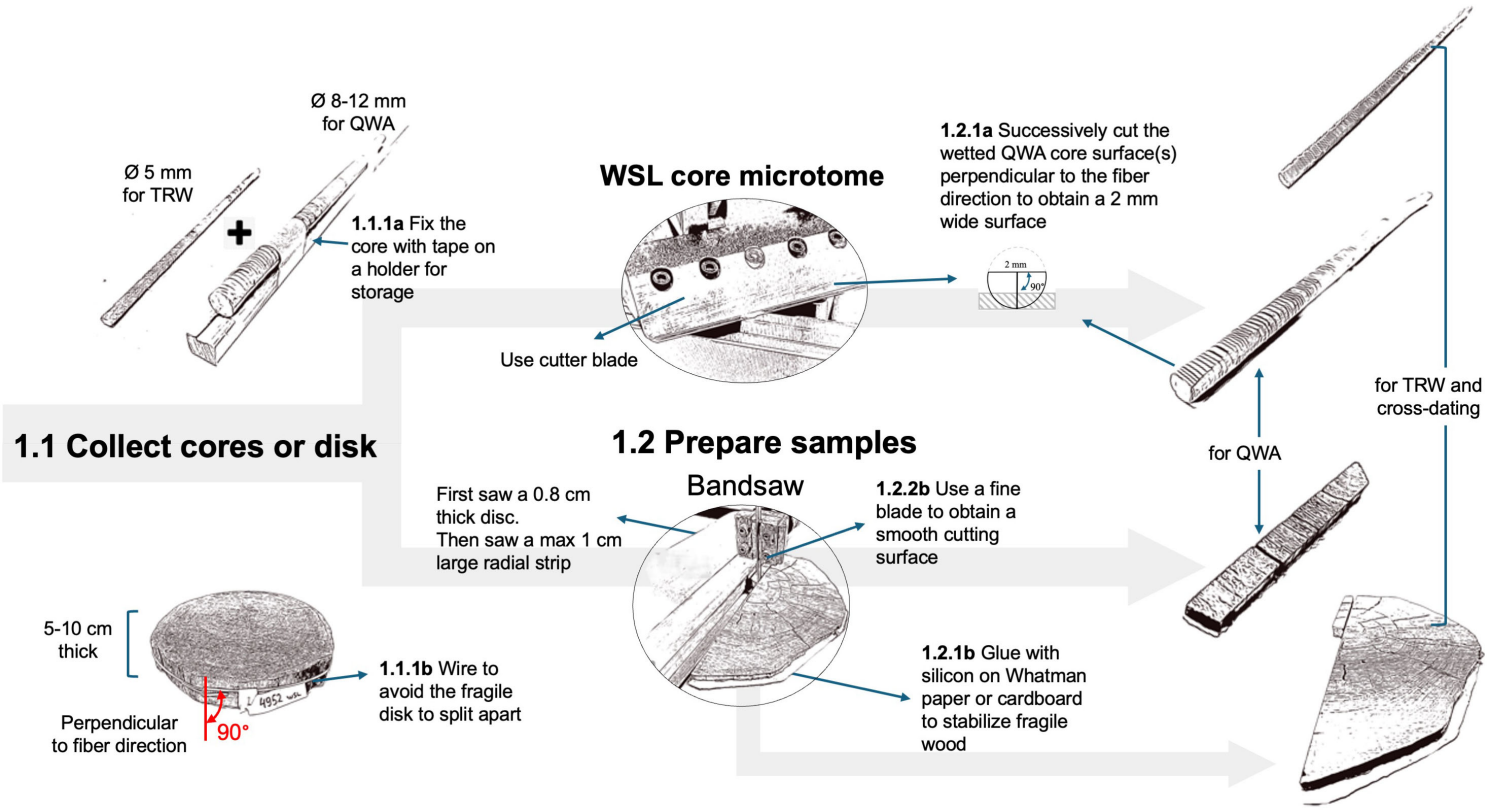
Workflow related to the collection and preparation of the samples. Two different paths are described depending on the form of the wood material collected (core or disk).

When collecting new wood material, it’s crucial to prioritize intact and properly oriented samples (**Figure 3**), ensuring that micro-sections can be taken perpendicular to the orientation of the conduits.

For obtaining cores, use well-sharpened increment borers (e.g., 3-Thread Increment Borers Haglöf, Sweden), preferably powered by a cordless drill (Metabo Cordless drill BS 18 LTX BL Q I with a PowerX3 torque setting adapter, Metabo, Germany) for constant rotation to reduce micro-cracks (Gartner et al., 2023). Opt for larger cores (8-12 mm in diameter) to increase the chances of obtaining untwisted and intact wood cores. Wrapping the cores in paper or inserting them in paper straws avoids molding, reduces bending, and allows the wood to shrink without cracks during drying. Avoid gluing cores onto wood holders, as glue entering the cell conduits will obstruct paraffin infiltration. For 5 mm cores, consider collecting an additional core in the same radial direction for tree-ring width measurements. Separating the tree-ring width measurement process will simplify the preparation of the anatomical samples.

When dealing with stem sections, e.g., from subfossil wood, prioritize selecting samples (cookies or wedges) with well-oriented cutting planes and of manageable sizes (i.e., about 5-10 cm thickness). Sufficiently thick stem sections facilitate the preparation of an adequate radial strip which should not be thicker than 1 cm in tangential width and longitudinal height to fit into the mold. For fragile wood, prevent uncontrolled splitting by gluing the surface of the wood disk on a thick paper sheet or cardboard using silicone glue (e.g., Bau-Silikon transparent, OBI, Switzerland) that will not infiltrate the cell conduits due to its viscosity. Ensure the radial strip is sawn perfectly perpendicular to the conduit’s longitudinal axis, preferably using a band saw (e.g., Hammer Bandsäge N2-35, Felder group, Switzerland) with 0.6 mm deep fine blades (e.g., FLEX-BACK-Sägeblatt Felder group, Switzerland) to maintain a clean-cut surface with unfilled lumina and reduce damage to the cell walls. Keep an adjacent radial strip for tree-ring width measurements and dating. Additional orientation adjustments can be made by sanding the sample if needed.

If not done previously, perform the tree-ring width measurements required for cross-dating. Preferably, perform these measurements on the additional adjacent sample collected earlier using traditional measuring systems, such as a microscope connected with a moving table, or using novel imaging-based approaches (e.g., https://youtu.be/8q78USQksXY) together with software such as CooRecorder (Maxwell and Larsson, 2021). However, if there is not enough wood available and tree-ring widths must be measured on the same core used for wood anatomy, measurements must be conducted without mutual interference. Therefore, tree-ring width measurements should be conducted on cut surfaces rather than sanded ones to facilitate infiltration through unobstructed cell lumina. This is best done on species with clearly distinguishable ring borders to avoid filling the conduits with chalk, and on untwisted cores where the fiber direction remains constant along the core. For both large (>8 mm) and standard (5 mm) cores, both the upper and lower cross-sectional surfaces can be cut using the WSL core microtome (Gartner and Nievergelt, 2010; Gartner et al., 2015) with disposable NT blades (Light-Duty A type NT BA-170) to facilitate paraffin infiltration. Ensure that no more than 1/3 of the core diameter is removed on either side, but enough to obtain a perfectly cross-sectional surface of at least ~2 mm tangential width with open conduits, and to keep the sample well-anchored within the paraffin block.

### 2.2 Removal of extractants, splitting, labeling, and orientation

Once the wood cores or the radial strips have been prepared, they still need to be cleaned from resins and other alcohol-soluble extractants, which might interfere with paraffin infiltration and the sectioning. The removal of extractants is achieved through a 24-hour Soxhlet extraction with 96% ethanol using a Soxhlet apparatus (standard 500 ml Soxhlet extractor with dimroth condenser, and 1000 ml round flask, e.g., Carl Roth GmbH + Co, Germany; with an electric heater, ELET36002-18, VWR International). It is important to note that ink-based labels on the sample might be washed out or hidden because the samples (especially if subfossil) may turn dark during the process. To avoid this, labels should be marked with a water-resistant soft pencil (e.g., Stabilo aquarellable All 8008, Galaxus), and a picture of the samples before the insertion in the Soxhlet extractor additionally helps label reassignment if needed. Silicon-glued paper can be kept on fragile samples since it does not interfere with the extraction process and the infiltration of paraffin in the tissue processor (see next steps).

A clear and consistent sample labeling strategy is crucial for effective wood processing and subsequent data analysis. Since wood samples are often split into sub-pieces for lab processing and imaging, and potentially further divided into sub-images depending on the analysis system used, it is essential to adopt a labeling system that can track the order and identity of sub-pieces and images throughout the entire workflow, from sample preparation to data analysis.

**Figure 4:**
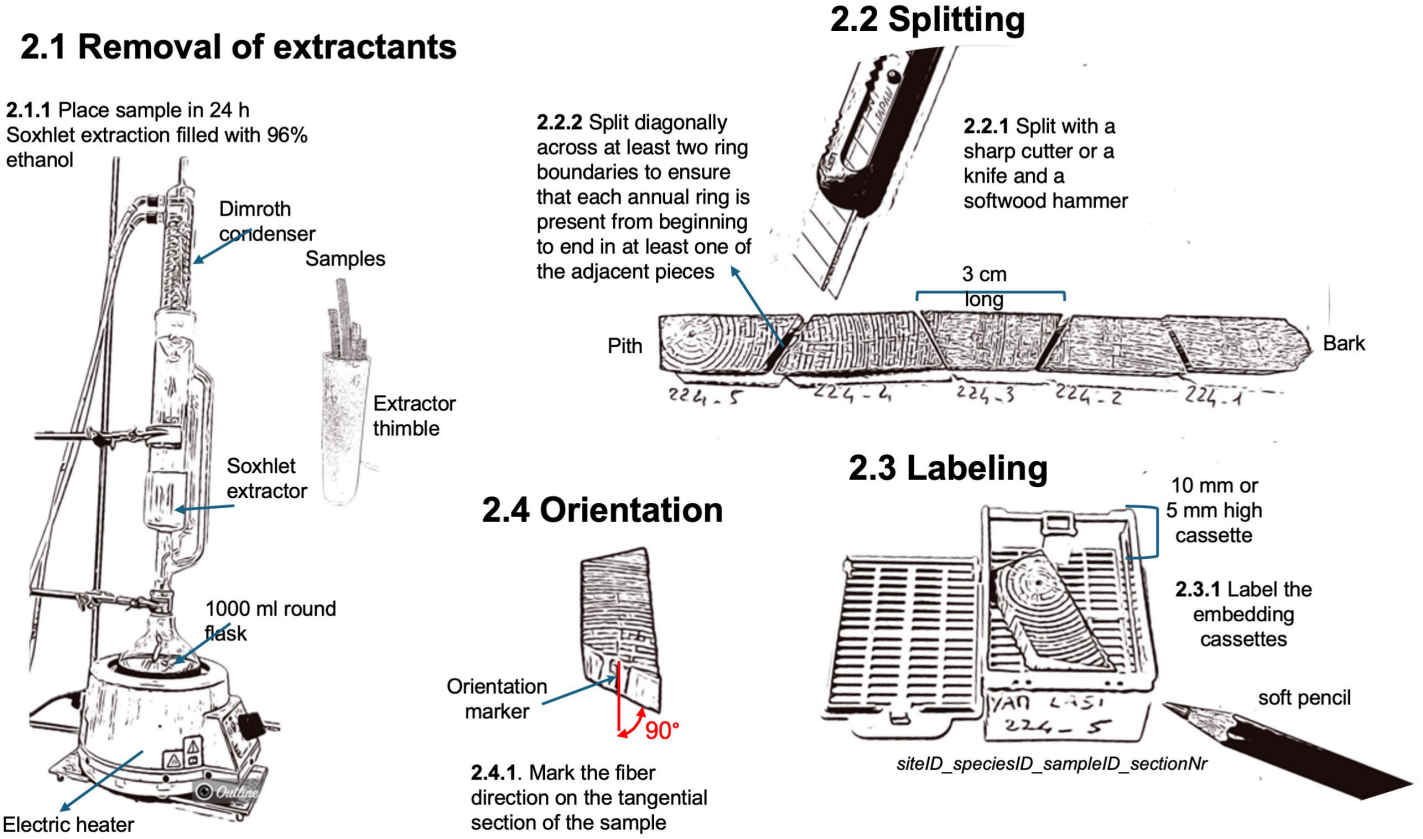
Workflow related to the removal of extractants, splitting, labeling, and orientation of the wood samples.

At the WSL laboratory, a standardized labeling system has been developed to facilitate tracking samples and their fragments during processing. This system ensures that the sample’s origin can be traced, even as the material is divided and imaged in successive stages. The labeling structure includes key elements separated by an underscore to simplify data handling: siteID_speciesID_tree/sampleID_sectionNr_imageNr (with siteID and speciesID being optional, depending on the study design). For example, the label “YAM_LASI_224_01_1” refers to the first image of the first thin-section closest to the bark from tree Nr. 224, a *Larix sibirica* from Yamal in northern Siberia. SectionNr and imageNr are added incrementally as the sample is processed, maintaining chronological order from bark to pith (**Figure 4**).

For both cores and radial strips, the length of the split sub-pieces should preferably be around 3 cm long to allow them to fit into the embedding cassettes (Simport Histocette M490 and Simport Macrocette M512, VWR International) and ensure enough margins for correct positioning within the mold for paraffin embedding (37 x 24 x 05 mm for 5 mm cores and 37 x 24 x 09 mm for big/deep sample, e.g., embedding dish, stainless steel, Biosystems, Nussloch, Germany). To ensure that each annual ring is present from beginning to end in at least one of the adjacent pieces it is essential to split the sample diagonally across at least two ring boundaries with a sharp cutter knife (e.g., NT Cutter A-300 RP) and a wooden mallet (e.g., LUX Holzhammer Comfort 70 mm, OBI, Switzerland). Additionally, marking the fiber direction on the tangential side of each sub-sample will ensure a reference for a correct orientation within the paraffin block. For cores with surfaces prepared on both the lower and upper sides, mark the side intended for sectioning.

### 2.3 Paraffin infiltration and embedding

**Figure 5:**
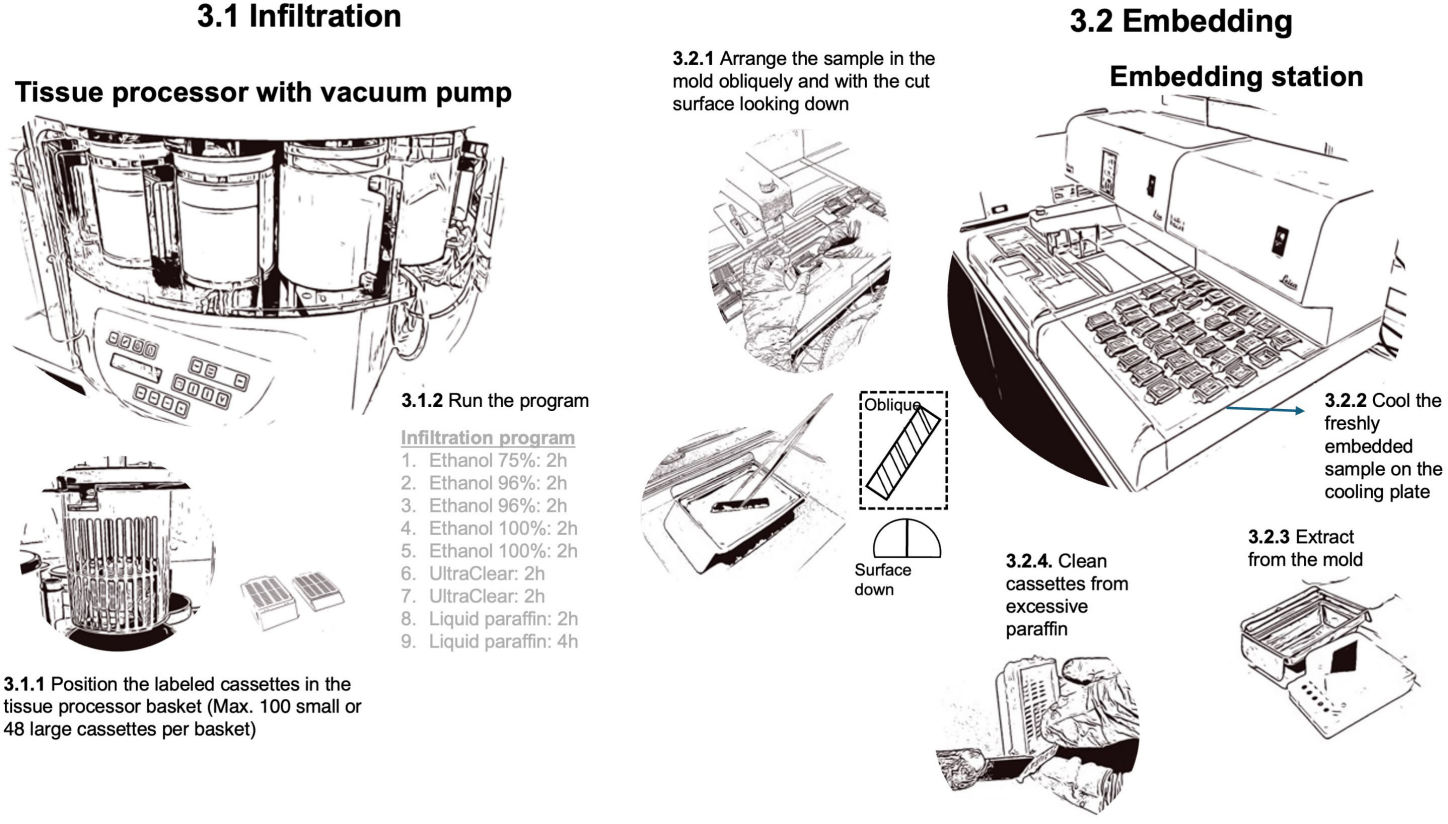
Workflow illustrating the paraffin infiltration and embedding process, with the duration of each step explicitly detailed.

Paraffin infiltration and embedding stabilize the samples during sectioning by filling all hollow spaces, ensuring that the physical dimensions remain intact throughout processing. To achieve complete infiltration, we first submerge the samples in water and place them in a vacuum chamber connected to a pump (e.g., 2 Gal (8L) Vacuum Chamber Kit, Shenzhen Haocheng Instrument Co., Ltd, China). We run 4 to 5 cycles of applying a vacuum in the chamber until the water starts to boil and then release the vacuum. To infiltrate woody tissues with molten paraffin (Paraplast™, melting point 56°C, Biosystems, Nussloch, Germany) we use a tissue processor (Leica TP 1020, Biosystems, Nussloch, Germany) (**Figure 5**). During this stepwise process, water is first removed from the tissue by increasing the purity of ethanol and subsequently, the ethanol is removed from the tissue using UltraClear (J.T Baker UltraClear™), a substitute and more environmentally friendly and less harmfull for humans clearing agent than the commonly used Xylene. Paraffin infiltration occurs as the samples are immersed in a bath of liquid paraffin, with a vacuum pump employed to enhance the rate of paraffin penetration into the tissues. Shaking additionally improves paraffin infiltration into the tissue. An example of the detailed infiltration program, including the duration of each step, is presented in **Figure 5**.

After tissue infiltration, the sample is embedded within a paraffin block to facilitate fixing it in the microtome holder and to further stabilize it during sectioning. This is achieved with an embedding station (e.g., Leica HistoCore Arcadia H, Biosystems, Nussloch, Germany), where the still-warm wax-infiltrated tissues are placed in the mold of previously labeled cassettes, which are then filled with warm molten paraffin. As the surrounding paraffin cools, it solidifies and provides a hard, stable, and solid block to support sectioning. To prevent the infiltrated samples from cooling faster than the surrounding paraffin and causing discontinuities (cracks) between the sample and the paraffin block, this process should be performed relatively quickly. If cracks occur, the sample embedding in the paraffin block should be repeated. Proper sample orientation in the mold is crucial, which is facilitated by the previous marking of fiber direction. If wood surface cutting was done perpendicular to the fiber direction, this will result in a parallel alignment of the transverse plane of the wood sample with the block surface for sectioning. Since the surface at the bottom of the mold will become the cutting plane, the sample plane selected for thin sectioning should be positioned facing the bottom of the mold. For silicon-paper-glued subfossil material, this means that the sample is positioned with the paper side facing up. Moreover, each specimen should be positioned so that the blade strikes it diagonally rather than parallel to the tree rings. This angle ensures that both the dense latewood and the less dense earlywood are cut simultaneously. This is usually achieved by placing the sub-sample obliquely within the mold. For the positioning press the sub-sample to the bottom of the mold with straight pointed tweezers (e.g., Tweezers straight pointed, 130 mm, Carl Roth GmbH + Co, Germany) until the paraffin starts to solidify. Large cassettes of 10 mm height, as used for the infiltration of large samples, should be replaced by equally labeled small cassettes (5 mm high) without lids to match the clamp size of the microtome and better stabilize the sample. To accelerate the solidification of the paraffin block, the embedded samples can be placed on a cooling plate (e.g., Leica HistoCore Arcadia H, Biosystems, Nussloch, Germany) for 20 minutes and/or stored in a regular refrigerator at ~ 4-6°C for 30 minutes until they can easily be extracted from the mold. Use abrupt and forceful pull to separate the paraffin block from the cassette.

After embedding, excess paraffin surrounding the solidified block on the sides of the cassette should be removed with a disposable blade (e.g., Light-Duty A type NT BA-170) to ensure a stable grip within the microtome clamp (universal cassette clamp 14 0502 37999, Biosystems, Nussloch, Germany).

### 2.4 Trimming

**Figure 6:**
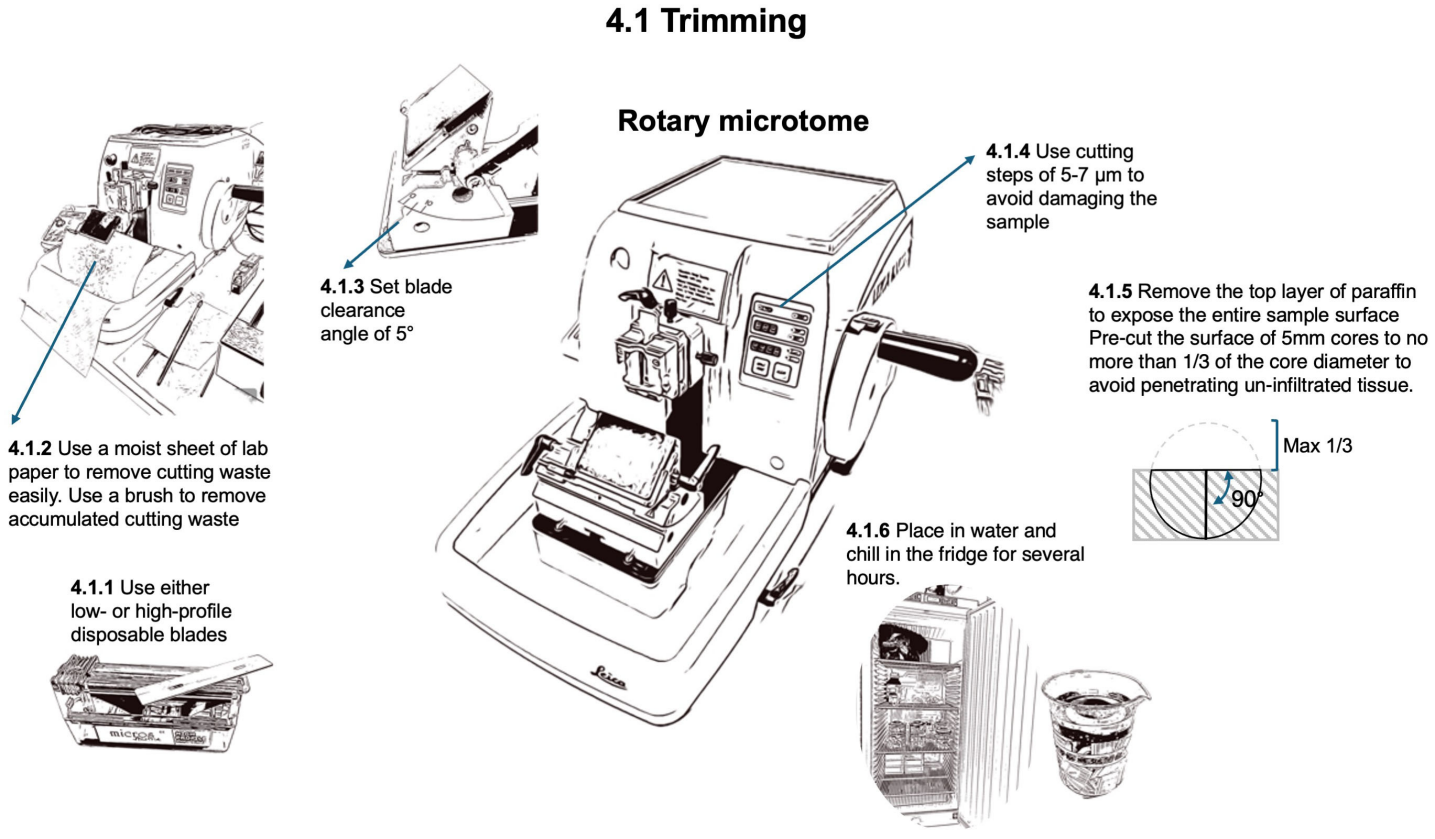
Workflow for trimming the block surface to expose the tissue at a level where a representative section can be obtained.

To prepare for sectioning (**Figure 6**), the wood surface of the sample needs to be exposed by removing the top layer of paraffin and wood. This process called trimming, precutting, or facing, is performed with the rotary microtome (HistoCore AUTOCUT, 14051956472, Biosystems, Nussloch, Germany) using either low- (FEATHER^®^ N35, Biosystems, Nussloch, Germany) or high-profile (Micros HP, Biosystems, Nussloch, Germany) disposable blades, with a cutting step of 5-7 µm to avoid damage to the dry and hard samples until the desired sample surface is entirely exposed. The primary difference between high-profile and low-profile disposable blades lies in their geometry and intended applications. High-profile blades are larger in height and have a steeper cutting angle, providing greater visibility and clearance, which is beneficial for broader or more aggressive cutting tasks. In contrast, low-profile blades have a flatter design, allowing for closer contact with the sample surface and offering finer precision, making them ideal for precision sectioning. The blade angle directly affects cutting efficiency and sample quality, with high-profile blades excelling in robust tasks and low-profile blades offering better control and stability for obtaining thin, high-quality sections for anatomical studies.

For trimming, set a blade clearing angle of 5°. The blade can be used as long as no artifacts such as stripes from notches in the blade are appearing. Standard 5 mm cores with unprepared wood surfaces before infiltration and embedding should be pre-cut to a depth not exceeding 1/3 of their diameter. This ensures that at least 2/3 of the core remains in the block, thus avoiding tissue that is not properly infiltrated by paraffin. To remove accumulated cutting waste from the blade holder, use a brush (Pelican Hair brushes no. 23, size 2-3) with bottom-up movements, i.e. away from the blade edge, to avoid trimming the brush hairs. To facilitate the cleaning of the microtome and the working place, place a moist sheet of lab paper at the bottom front of the microtome to easily remove the waste generated at the end of the trimming process.

Before thin sectioning, it is recommended to immerse the samples in water and store them at 4-6 °C in the refrigerator for several hours (preferably overnight) to harden the paraffin and soften the wood. These conditions are necessary to ease sectioning and prevent the paraffin from sticking to the microtome blade holder or brush, thereby enabling the creation of a cohesive “ribbon” of subsequent thin sections.

### 2.5 Sectioning and floatation

**Figure 7:**
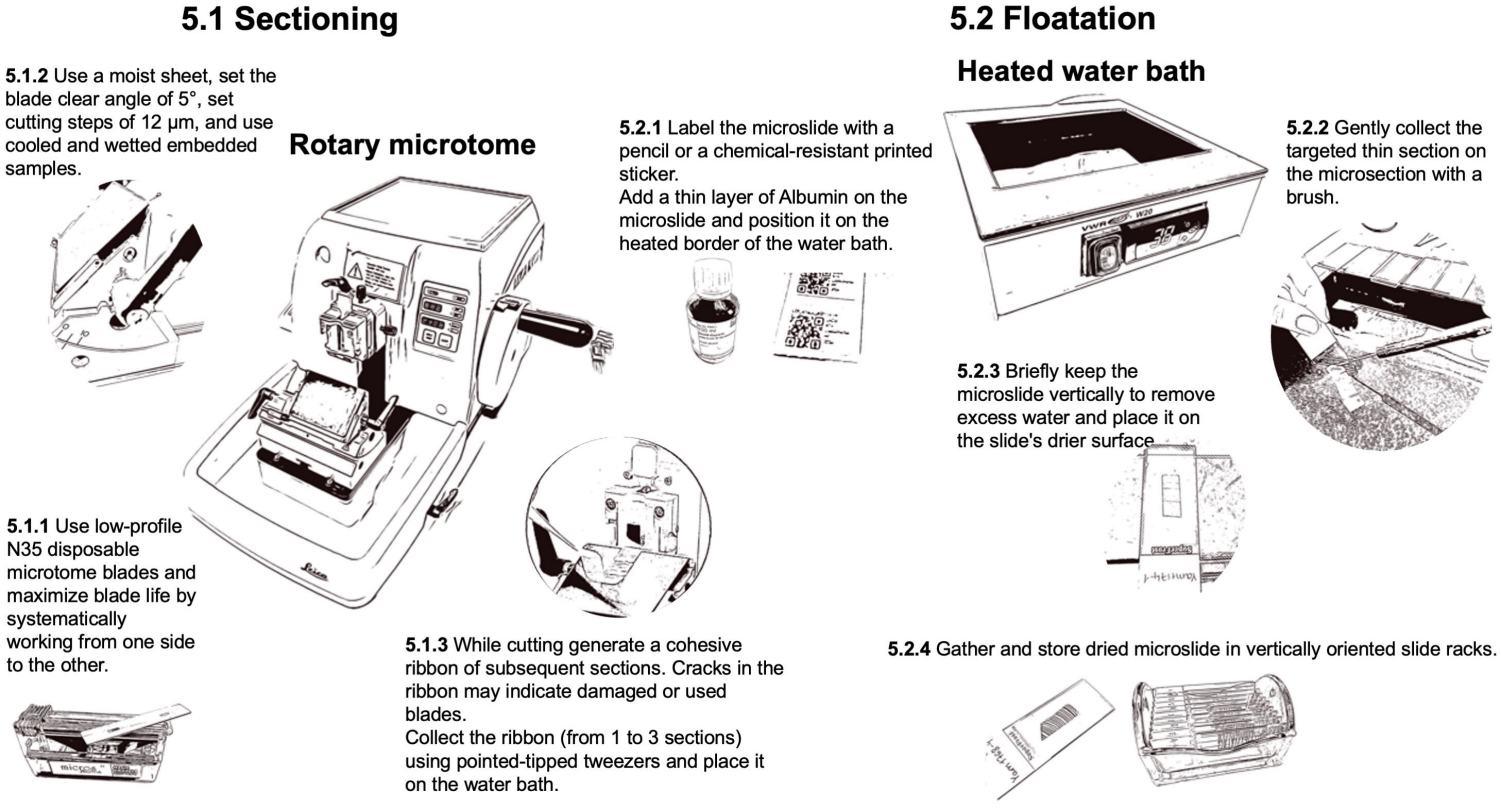
Workflow for the preparation of thin-section with a rotary microtome.

For sectioning (**Figure 7**), low-profile N35 disposable microtome blades (FEATHER^®^ N35, Biosystems, Nussloch, Germany) are typically used. In some cases as for denser woods, more stable high-profile blades may be preferred (Micros HP, Biosystems, Nussloch, Germany). The typical blade wear is 10 samples. Set the blade clearing angle at 5°. To maximize the blade usable duration, move the blade systematically, working from one end to the other. The ideal section thickness for achieving optimal staining intensity and contrast in QWA measurements (e.g., using ROXAS) is 12 µm. Thicker sections (i.e., 14-16 µm) are not recommended due to their greater weight, which increases the likelihood (up to 70%) of losing the section during the subsequent staining process. While sectioning, ribbons of generated sections are produced and collected. If the ribbon does not form well, ensure that the block is still sufficiently cold. Cracks in the ribbon may indicate damaged or used blades. Even slight contact with metallic items, such as the tweezer tips or the ferrule (metal band) of the brush, can damage the blade surface. Hard impurities in the sample can also cause these cracks. Moving or changing the blade, as well as flipping the sample in the microtome clump, may resolve any issues.

To collect the selected sections from the ribbon (from 1 to 3 sections), use pointed-tipped dissection needle (e.g., Dissecting needle straight, Carl Roth GmbH + Co, Germany) to first transfer the selected ribbon floating on top of a 38°C water bath (VWR^®^ W20, Histology Water Bath, VWR International) with the smooth (shiny) side down. The warm water helps the floating ribbon (floatation) to stretch and ensure it is completely flat. Examine each section as it floats and inspect for imperfections when selecting the target section. The target section can then be caught and positioned on a pre-labeled and thinly albumin-coated microscope slide (SuperFrost^®^ slides with 90° ground edges, Biosystems, Nussloch, Germany) by holding it with a brush while gently lifting the slide from below the floating section. Therefore, one drop of Albumin (Protein glycerol, P049.1, Carl Roth GmbH + Co, Germany) is applied to the slide surface with a gentle single wiping movement to obtain a homogeneous thin layer (without gaps or excess), which keeps the thin section sticking to the slide during the staining process. The slides should also be labeled accordingly with a pencil or a chemical-resistant printed sticker (Labid-technologies N0FTT-149C1-2WH or Biosystems 85-1086-00, Nussloch, Germany; printed with the Zebra ZD621 label printer using the Zebra ZD621 software, Zebra online store). If the slide scanner supports it, consider using QR codes for labeling. Don’t use solvable ink to prevent the labels from fading or dissolving during the staining process. Additionally, as the warmed paraffin has become sticky, the brush should only be very gently held on the sample during the collection of the target section to prevent damaging the thin section. Once the target thin section adheres to the slide, drain them vertically for a brief time to remove excess water and place the slide on the slide drier surface of the water bath until all water has evaporated. The slides can then be gathered in slide racks (glass rack for 10 slides with metal handle and staining jar, VWR International). Slides with thin sections can be stored in slide racks or slide boxes (Slide Boxes with Lid, VWR International) until staining. The samples in the paraffin blocks can be archived in an embedding carton box (Diastore Block 12, Histocom, Switzerland) and thus remain available in case the procedure needs to be repeated.

### 2.6 Dewaxing, staining, and fixing

**Figure 8:**
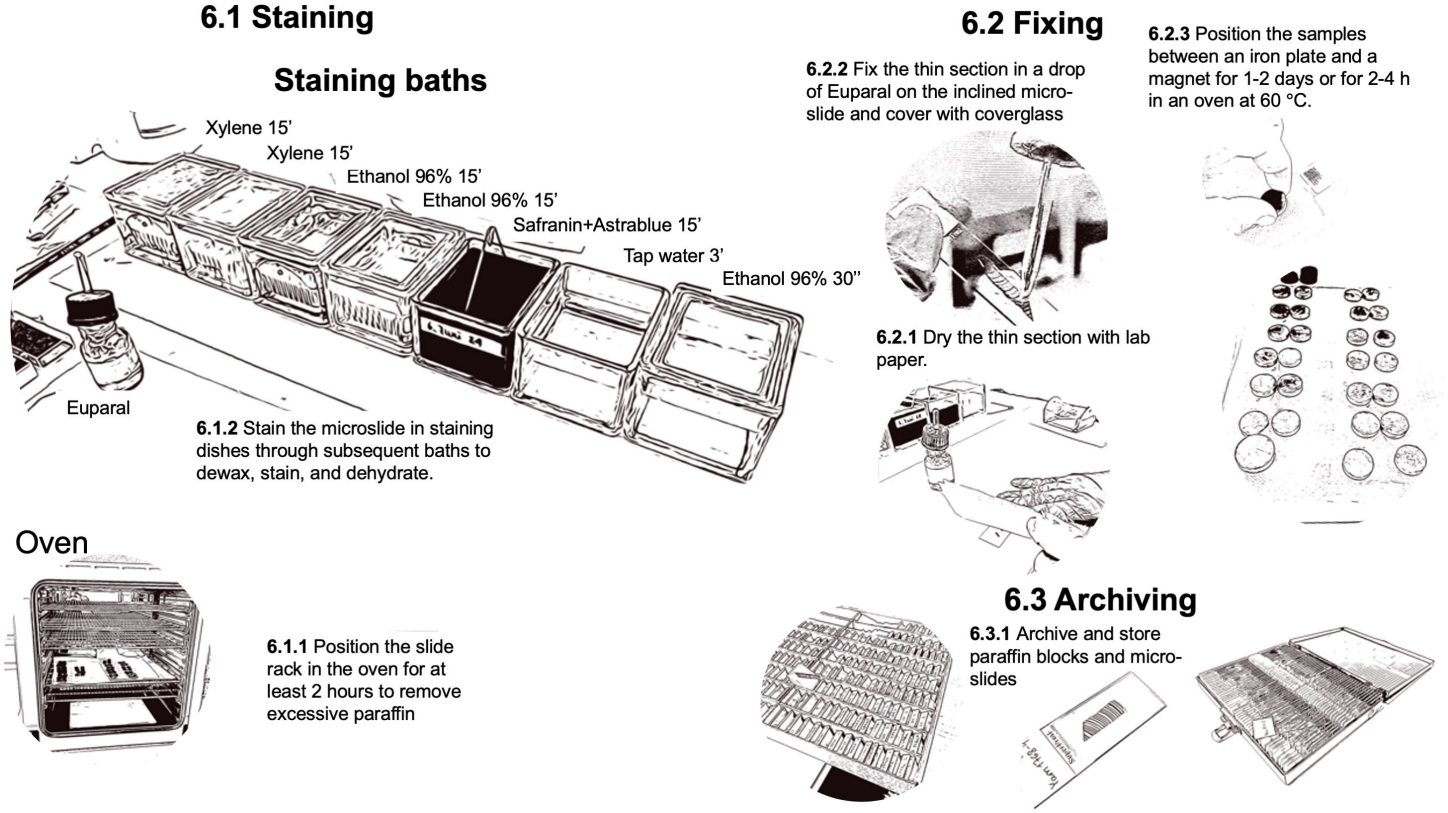
Workflow for the steps related to dewaxing, staining, and fixing of the prepared thin-sections.

To complete the process, the thin sections must be deparaffinized and stained to reveal the underlying tissue structures and facilitate quantitative image analysis (**Figure 8**). Additionally, they must be permanently fixed for secure handling, such as imaging and long-term storage. To melt and remove the paraffin from the prepared slides, place the thin sections in slide-racks and put them in an oven (BINDER FD 115 drying and heating chambers with forced convection, Faust, Switzerland) at 60°C for at least 2 hours before staining but no longer than 24 hours.

The staining process is performed under a fume hood with slide-racks in staining dishes using a mixture of 0.8% Safranin and 0.5% Astrablue [according to (Gärtner and Schweingruber, 2013)] to stain the lignin red and the cellulose blue. The process consists of subsequent baths to dissolve and remove the remaining paraffin with Xylene, to remove the Xylene with Ethanol, and to stain the thin sections. After staining, the samples are rinsed with water, dehydrated with Ethanol, and then prepared for fixation with Euparal (Euparal 7356.1, Carl Roth GmbH + Co, Germany). A staining protocol with relative bath durations is provided in **Figure 8**. The process can be performed in sequence with multiple slide-racks. The bath durations have been optimized according to the thickness of the thin section to guarantee sufficient contrast for further image analysis.

Sample fixation occurs immediately after staining. To fix the thin sections in Euparal, carefully dry the thin section after dehydration in 96% ethanol by gently patting the slide with a paper towel. Then, apply Euparal on top of the thin section using a pipette (Pasteur pipettes without cotton plug with bulb, Carl Roth GmbH + Co, Germany). For large thin sections covered by a 50 mm long cover glass (Cover Slips, VWR International), use two large drops. To reduce air bubbles, the applied Euparal drops should be allowed to flow down the inclined thin section and cover it completely before placing the cover glass. Fixation is achieved by placing the slide between a magnet (e.g., Ferrite magnets Y35 20mm diameter 5mm height, Supermagnete) and an iron plate (e.g., Ferrous metal plate, DIY/Home improvement store). Additionally, one could use a layer of polyethylene (e.g., LDPE Tubular film 200mm diameter 100µm thickness, Elke-Plastic, Switzerland) to protect the iron plate and magnets from the excessive fixing agents. Leave the slides in a laboratory fume hood for one to two days before removing the magnets. The process can be accelerated by putting the magnet-covered slides for 2-4 hours in a regular oven at 60°C. After drying, and before imaging, clean the excessive fixing agent with 96% ethanol to remove any potential dirt obstructing the imaging. If large quantities of fixing agents cover the cover glass, first use razor blades (Single Edge Stainless Steel Teflon Coated Blades, Biosystems, Switzerland) to carefully remove it, ensuring that the generated dust is also removed. If this repeatedly occurs, reduce the fixing agent applied. Since the staining and fixing process for an entire batch takes several hours, it is advisable to schedule this procedure on a day separate from sample sectioning.

### 2.7 Imaging

**Figure 9:**
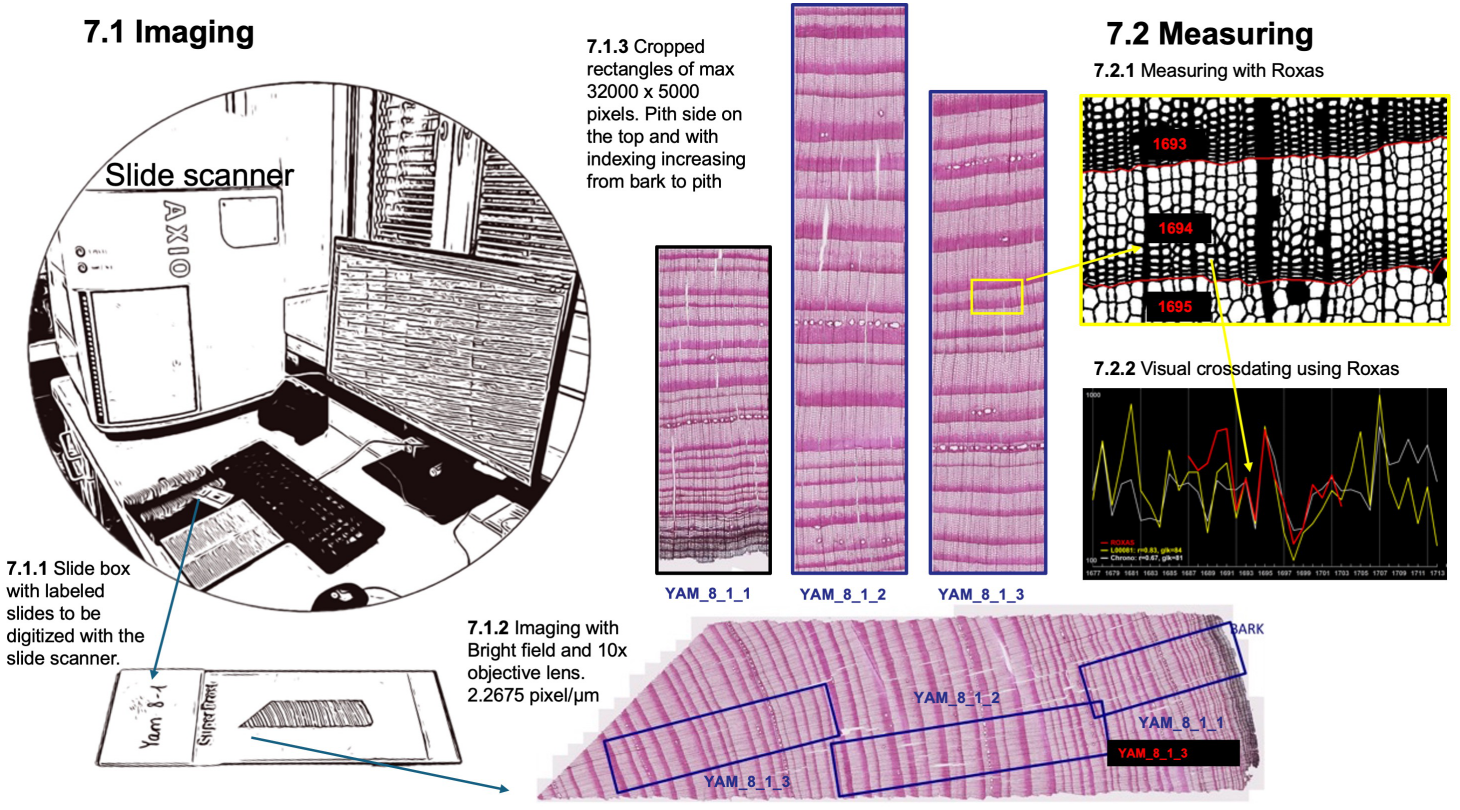
Imaging of the thin section and preparation of manageable sub-images for ROXAS measuring.

The fixed, labeled, and cleaned slides are prepared for imaging using a high-volume automated slide scanner (Zeiss Axio Scan.Z1 slide scanner, Carl Zeiss, Germany) (**Figure 9**). This advanced equipment efficiently acquires high-quality images of entire thin sections under consistent and controlled conditions. Specifically, bright-field imaging is employed with a 10x objective lens, maintaining constant illumination for a high resolution of 2.2675 pixels per micron. After producing a 3-dimensional map of the section surface to account for unavoidable waviness using autofocus, the entire section is digitized with a z-stack around the surface map and stitched to a flat image, thus ensuring sharp image quality. Slide IDs can be read directly by the system from the QR code on the label, provided by a text file list, or manually adjusted after scanning. For alternative imaging methods using common optical microscopes, including guidelines for image capture, focusing, illumination, and stitching, we refer readers to established protocols presented in von Arx et al., (2016). This reference details step-by-step recommendations to ensure high-quality imaging, even in the absence of slide scanners, as well as software tools for stitching images when scanning entire sections.

To ensure compatibility with ROXAS software for cell anatomical measurement, the collected images of thin sections undergo a specific processing procedure. A custom macro is used to place overlapping cropping rectangles on the whole-section image, ensuring each tree ring in the overlapping regions is fully represented in only one rectangle. This strategic placement selects the best-quality parts of the sections, avoiding issues such as cracks and disrupted tissue. The cropping rectangles adhere to defined dimensions, with a maximum length of 32000 pixels along the radial tree stem axis and approximately 5000 pixels in width (around 2.2 mm of wood) along the tangential axis. JPG images are then batch-exported as defined by the cropping rectangles, with the pith side oriented at the top. To maintain a clear hierarchical organization, the newly exported JPG images are labeled by appending an incremental number (ImageID) to the thin section label (cf. step 2). This numbering system progresses sequentially from the bark to the pith, facilitating subsequent analysis.

## 3 Troubleshooting

Because of the complexity of the entire process, the quality of the samples, and the natural variability of the sample wood quality, it is possible for the procedure to fail. However, common issues may arise, each of which has a potential solution. A summary of the more common issues and associated troubleshooting can be found in **Table 1**.

**Table 1:** Common issues and recommended troubleshooting when preparing thin sections.

**Microcracks**	**SAMPLE COLLECTION AND PREPARATION**
**Description**: Small fissures typically running parallel or oblique to the growth rings of the wood. These cracks typically leave cells intact, but affect cell position and don’t allow wall thickness measurements adjacent to the cracks. **Causes**: Microcracks often result from mechanical stress during manual coring. This can occur due to unstable borer guidance when inserting the borer into the trunk or from using a dull borer. **Prevention**: Use a sharp borer, ideally powered by an electric drill, to ensure smooth and consistent friction. Employing a 10 mm thick borer can reduce the likelihood of microcrack formation, as it provides more core stability during collection and allowing for the collection of wood sections further from the core surface, which are less likely to be affected by cracks. **Remedy**: None; use affected sample for measurements with caution.	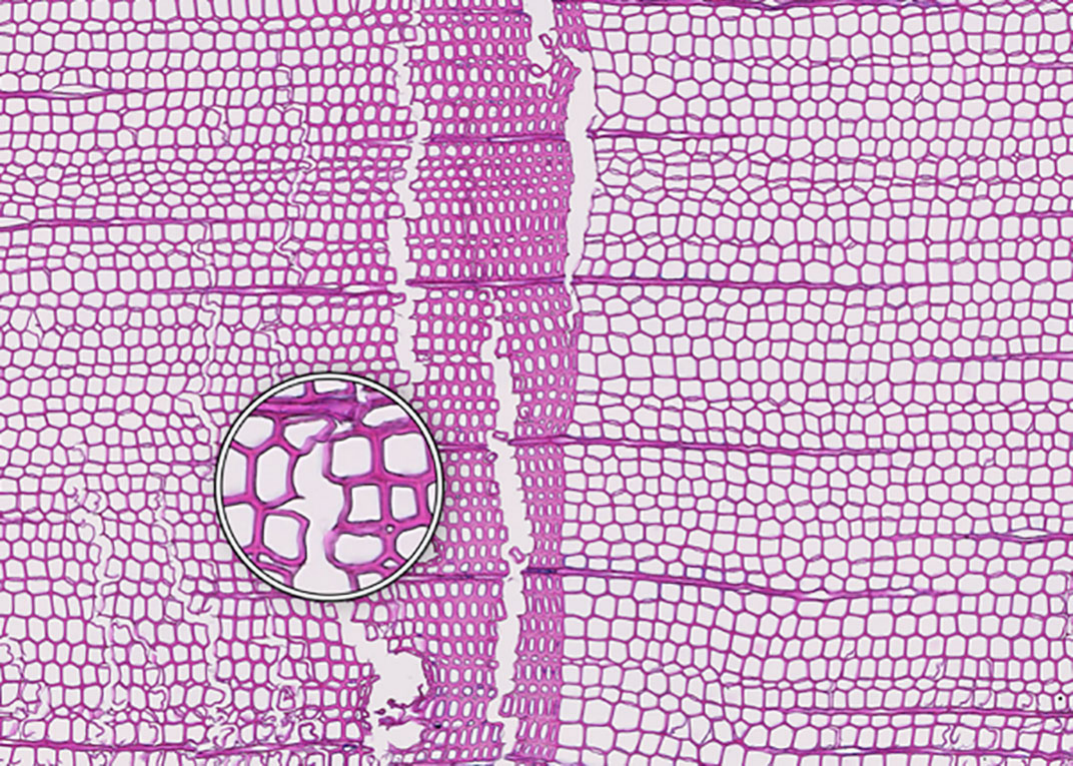 *Picea engelmannii*, T.W. Daniel Experimental Forest, Utah (USA), credit: Matt Bekker
**Microorganisms**	**SAMPLE COLLECTION AND PREPARATION**
**Description**: Thin section characterized by the presence of microorganisms, such as fungi or bacteria, within the wood tissue. These organisms may appear as dark spots, irregular structures, or hyphal networks, often disrupting the normal cell structure and resulting in less intense staining in the affected areas. This disruption can complicate the identification of the cells. **Causes**: Microbial infiltration typically results from wood decay or contamination, often occurring in compromised or decaying wood. **Prevention**: Collect samples from healthy wood and ensure they are stored under dry conditions. **Remedy**: None; heavily affected samples should be excluded from analysis, as they may yield unreliable data.	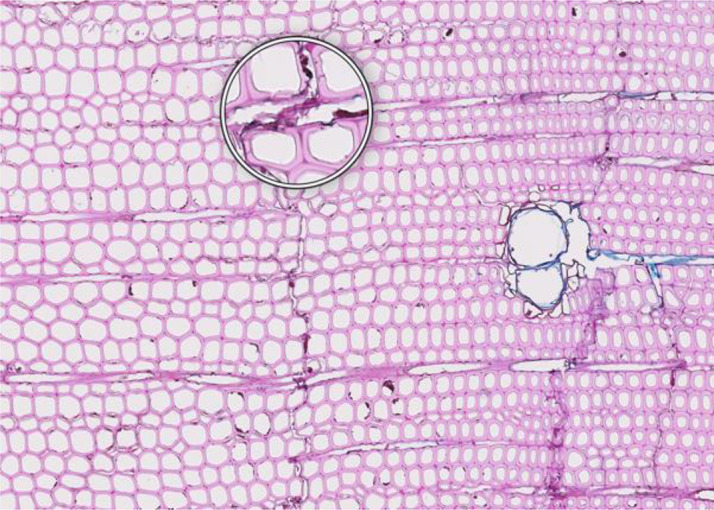 *Pinus pinea*, Central Spain, credit: Macarena Ferriz Nuñez
**Cell walls broken in random directions**	**SAMPLE COLLECTION AND PREPARATION**	
**Description**: Thin section with tracheid walls broken or disrupted in different directions. This damage prevents the correct recognition of the cells. **Causes**: Broken cell walls in all directions often result from an inadequate surface preparation, particularly sanding. This improper preparation can obstruct effective paraffin infiltration, leaving visible signs of sanding and contributing to structural weakness during sample sectioning. **Prevention**: Use a fine-toothed saw for extracting radial bars from larger wood pieces like stem discs. Smooth and level the surface of increment cores and radial bars with a core microtome (instead of sanding) to enhance paraffin infiltration. Remove enough surface wood with a rotary microtome to avoid damaged cells before thin sectioning. Ensure thorough paraffin infiltration by vacuuming the sample immersed in water beforehand. **Remedy**: None; utilize an area of exclusion if the affected region is relatively small.	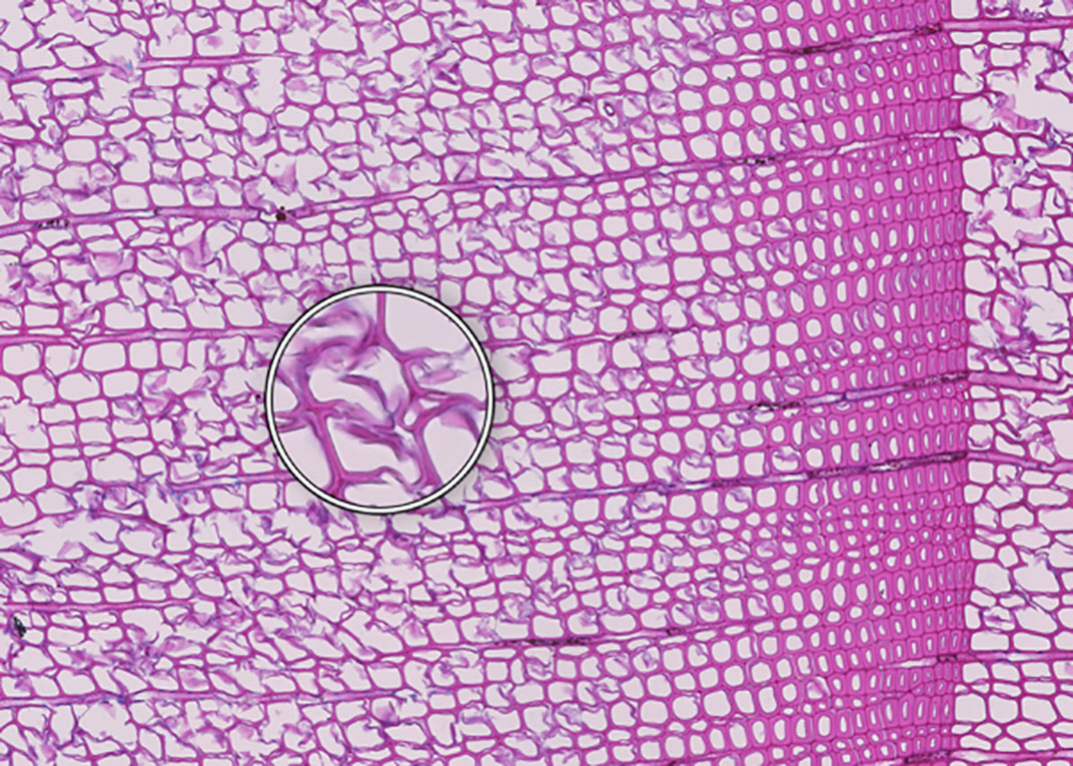 *Abies marocana*, Tazaot (Northern Morocco), credit: Patrick Fonti
**Red lumen**	**SAMPLE COLLECTION AND PREPARATION**
**Description**: The thin section shows lumina that are partly filled with red-stained inclusions that can prevent the recognition of cell structures. **Causes**: This issue is likely caused by the deposition of gums or other substances into the affected lumina. **Prevention**: Such depositions may be species-specific and often don’t disappear even after Soxhlet extraction. There might be wood samples that are less affected. **Remedy**: None; use affected sample for measurements with caution.	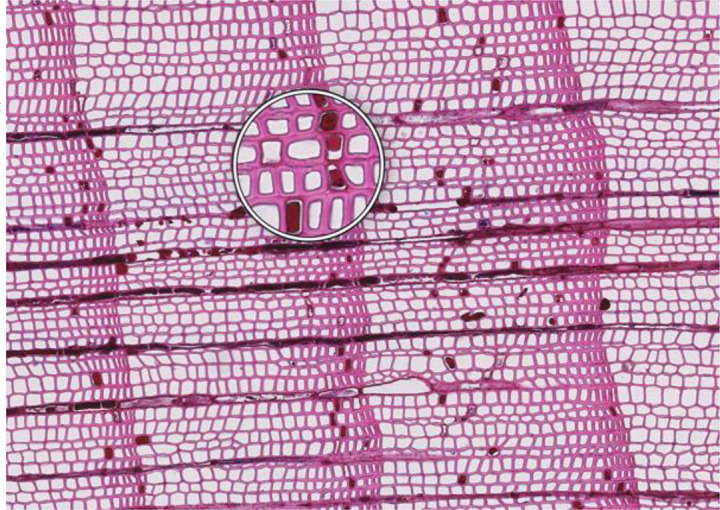 *Fitzroya cupressoides*, Rio Alerce (Argentina), credit: Marina Fonti
**Compression wood**	**SAMPLE COLLECTION AND PREPARATION**
**Description**: The thin section shows compression wood, characterized by abnormal cell structures and density changes that can affect the overall analysis of the wood sample. This issue is different from all above as there might be no technical problem, but rather an unfortunate choice of wood material. **Causes**: Compression wood typically develops in response to mechanical stress or gravitational forces, leading to altered growth patterns and irregular cell wall formation. **Prevention**: Select compression wood free areas or samples from stable and healthy trees. **Remedy**: None; don’t use affected samples or tree rings for measurements, unless these structures are the target of the investigation.	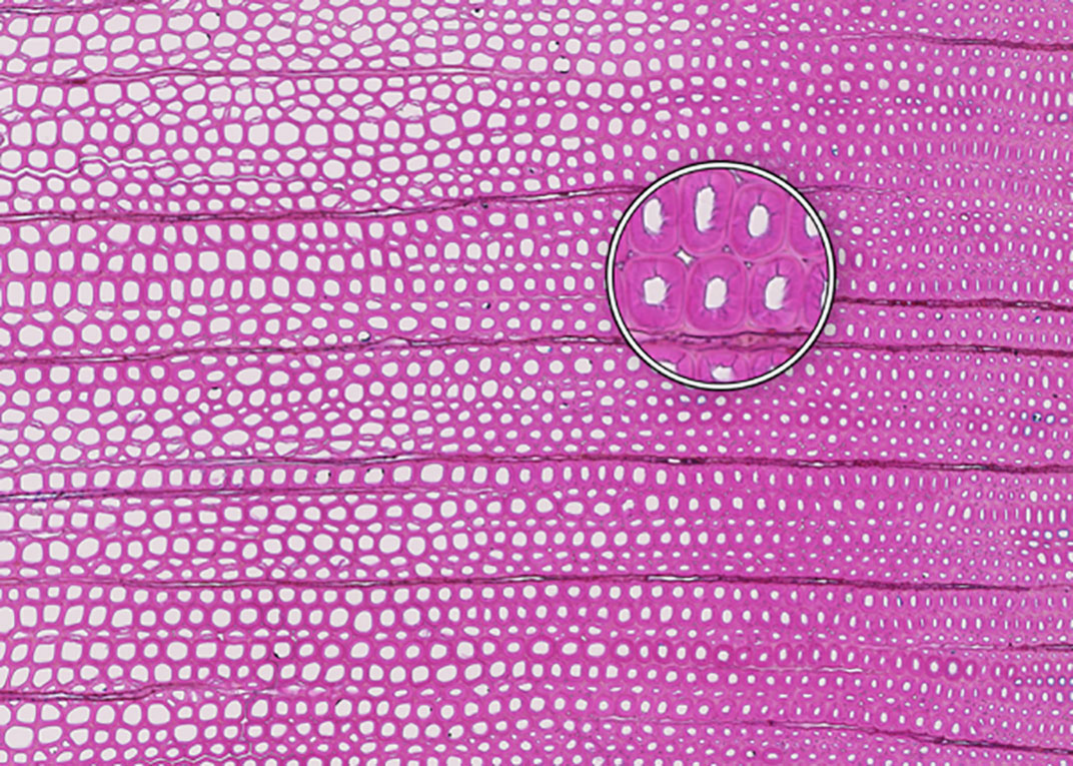 *Larix sibirica*, Yamal (Siberia), credit: Marina Fonti
**Cell walls directionally disrupted**	**PARAFFIN INFILTRATION AND EMBEDDING**
**Description**: The thin section is characterized by disruptions in the cell wall that all point in the same direction, which can hinder cell measurement. **Causes**: This type of broken cell wall often results from insufficient paraffin infiltration during embedding. This insufficient infiltration can occur if the sample surface has not been properly prepared or if the sample has not been adequately vacuumed, leaving weak spots that are susceptible to fracturing when the microtome blade strikes the tracheids. **Prevention**: Section the wood surface to enhance paraffin infiltration, ensuring that the sections are deep enough. Additionally, vacuum the sample immersed in water before paraffin infiltration to promote thorough infiltration. **Remedy**: None; utilize an area of exclusion if the affected region is relatively small.	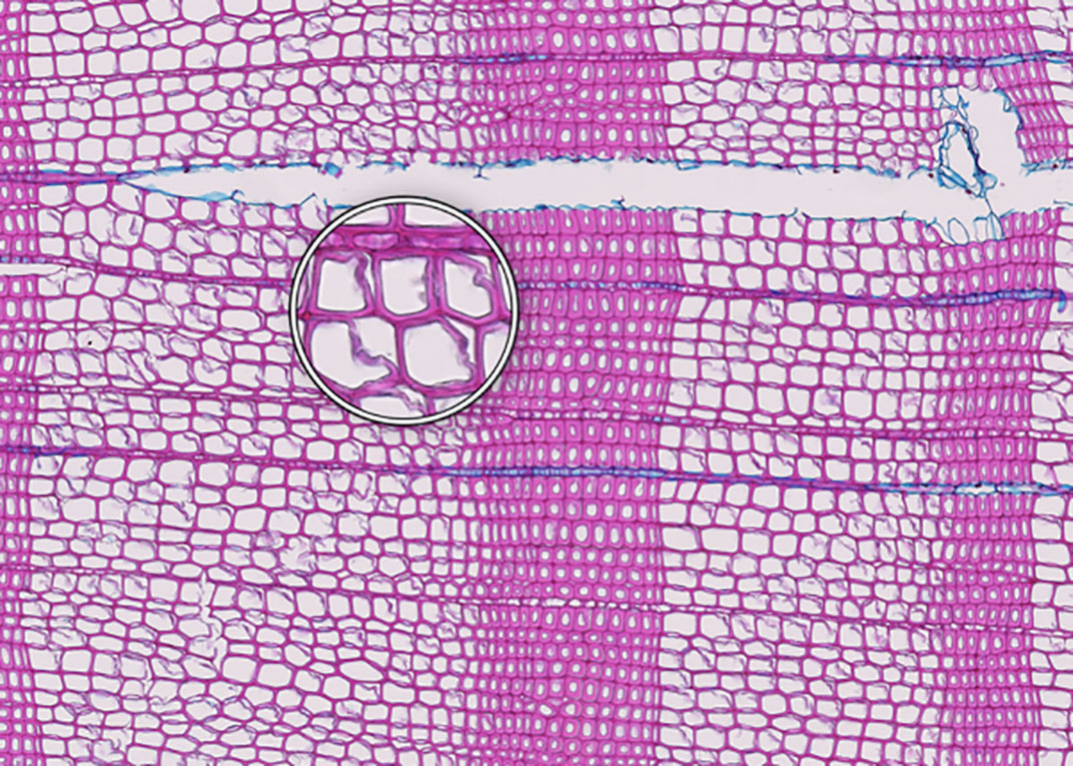 *Pinus sylvestris*, Gotland (Sweden), credit: Marina Fonti
**Fuzzy lumen outlines**	**PARAFFIN INFILTRATION AND EMBEDDING**
**Description**: The thin section shows unsharp cell lumina, indicating that tracheid orientation was not orthogonal during sectioning, leading to a distorted appearance and underestimated lumen area. Tangential misalignment causes pits to appear in a side view, while radial misalignment makes rays appear shortened across the section. **Causes**: Fuzzy lumina typically arise from improper sample positioning during the embedding process. **Prevention**: Ensure correct sample orientation during both sample collection and embedding to avoid misalignment. **Remedy**: Re-embedding	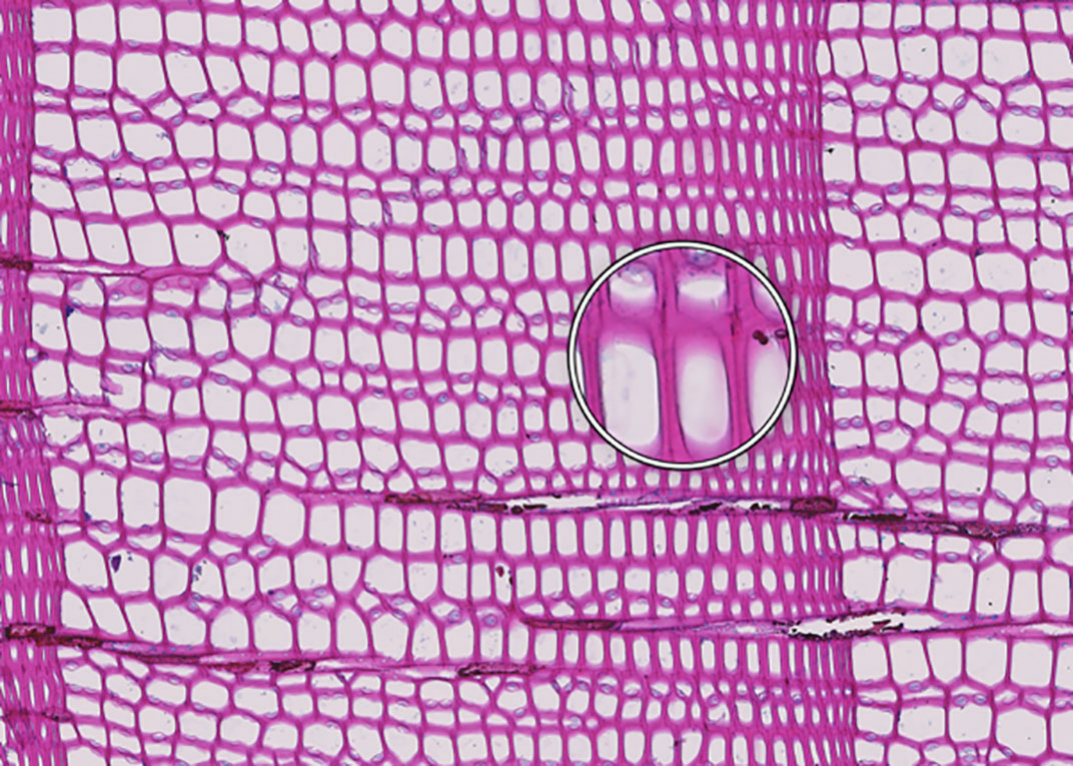 *Cedrus atlantica*, Tazaot (Northern Morocco), credit: Daniel Nievergelt
**Cell walls broken along lines**	**SECTIONING AND FLOATATION**
**Description**: The thin section is characterized by the presence of broken tracheids and cracks along lines. This prevents accurate measurement of the disrupted cells. **Causes**: These breaks often result from using a blunt or damaged microtome blade during sectioning. Additionally, hard particles within the sample that are pushed through the sample during sectioning can result in similar damage. **Prevention**: Use a sharp blade for sectioning to ensure even sections. **Remedy**: None; utilize an area of exclusion if the affected region is relatively small.	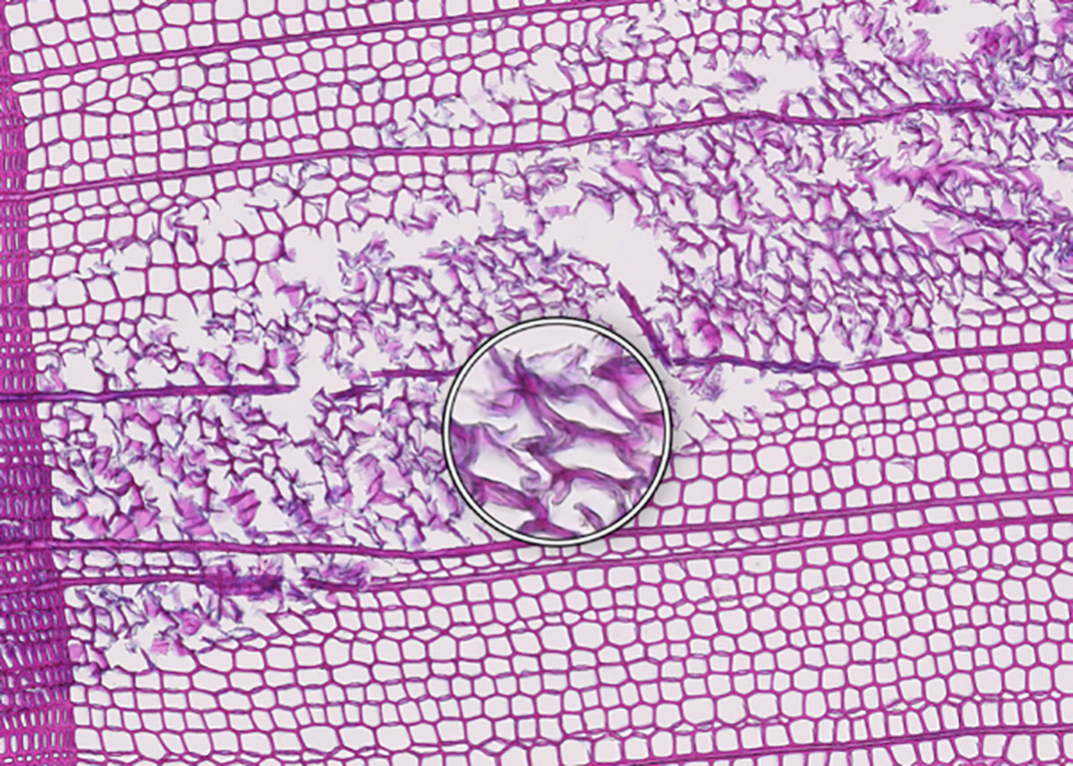 *Picea engelmannii*, T.W. Daniel Experimental Forest, Utah (USA), credit: Matt Bekker
**Light streaks**	**SECTIONING AND FLOATATION**
**Description**: Light streaks are characterized by uneven sections in the wood sample, leading to varying tissue thickness or minor cell wall disruption. This can introduce biases in measurements, affecting the accuracy of the data. **Causes**: This issue is typically caused by an improperly fixed microtome blade or poorly fixed samples, causing the blade to lose contact with the sample during sectioning. Additionally, cracked paraffin blocks can contribute to this issue. **Prevention**: Ensure the microtome blade is sharp and securely fixed, that the sample is properly clamped, and that the paraffin block is intact. **Remedy**: None.	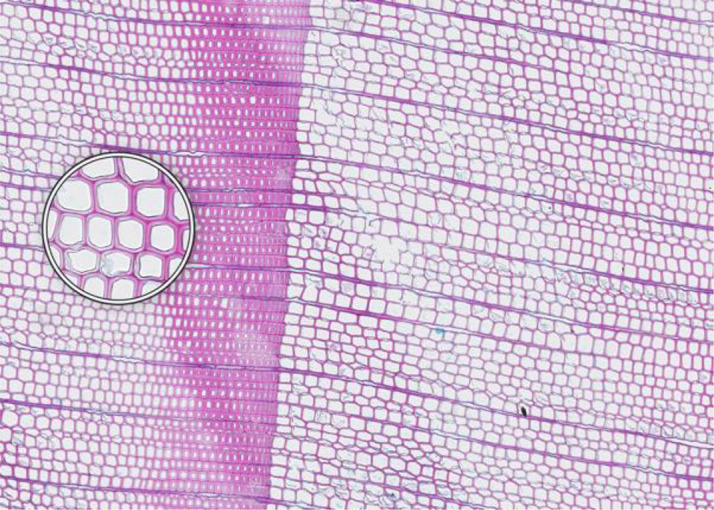 *Picea abies*, Croda da Lago (Italy), credit: Lenka Slamova.
**Folded tissue**	**SECTIONING AND FLOATATION**
**Description**: The thin section is characterized by overlaying tissues, typically occurring at the transition between earlywood and latewood. This overlap prevents correct cell recognition and thus results in locally wrong measurements. **Causes**: Overlaying tissues often result from density differences between earlywood and latewood, causing instability during sectioning. Improper tape sliding or handling in the water bath can also lead to tissue folding. **Prevention**: Reversing the sample orientation in the microtome clamp can reduce pressure from density differences. Proper tape sliding and careful handling in the water bath further minimize folding. **Remedy**: None; utilize an area of exclusion if the affected region is relatively small.	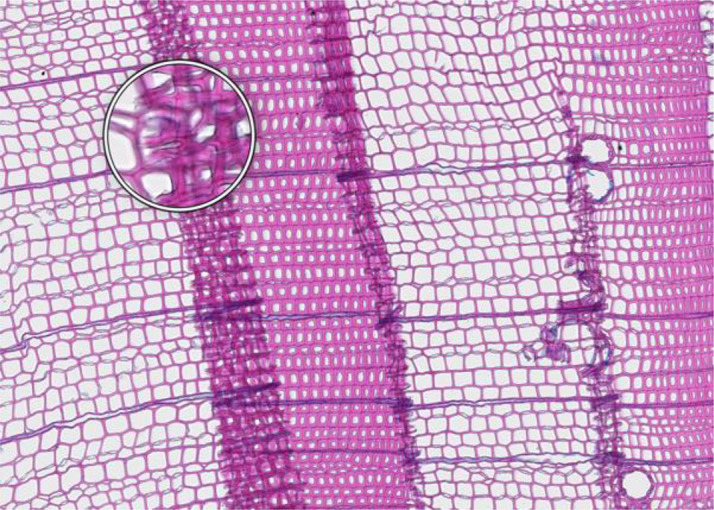 *Picea abies*, Kazitiskis (Lithuania), credit: Lenka Slamova
**Overlying tissue fragments**	**SECTIONING AND FLOATATION**
**Description**: The thin section shows overlying tissue fragments, where one tissue layer obscures underlying structures, preventing correct cell recognition and resulting in locally wrong measurements. **Causes**: Overlying tissue fragments often results from contamination due to a dirty microtome sliding table or water bath during sectioning. **Prevention**: Ensure cleanness of both the microtome and water bath to maintain optimal sectioning conditions. **Remedy**: None; utilize an area of exclusion if the affected region is relatively small.	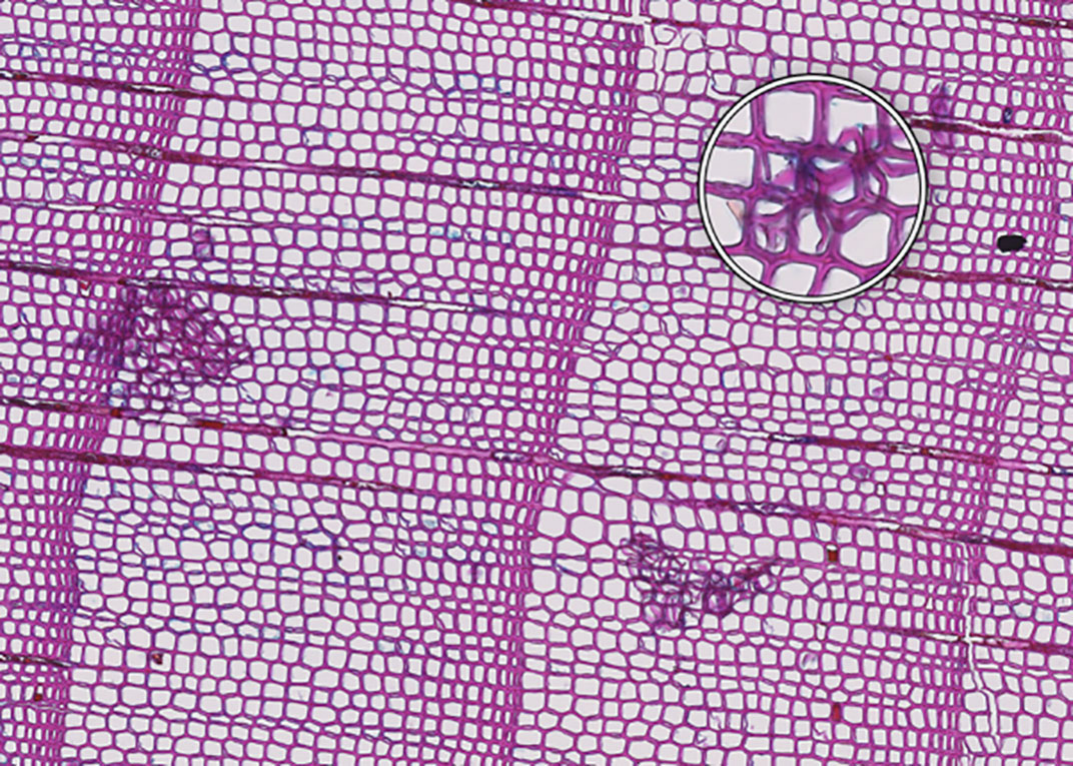 *Larix sibirica*, Yamal (Siberia), credit: Marina Fonti
**Albumin residue**	**DEWAXING, STAINING AND FIXING**
**Description**: The thin section shows the presence of albumin residue, appearing as a bluish smeared layer behind the tissue. If it is too dark, it may impede correct cell detection. **Causes**: Albumin residue typically results from an excessive layer of albumin on the slide, leaving protein traces on the wood surface. **Prevention**: Ensure that the amount of albumin applied to the slide is appropriate and evenly distributed. **Remedy**: None; use affected sample for measurements with caution.	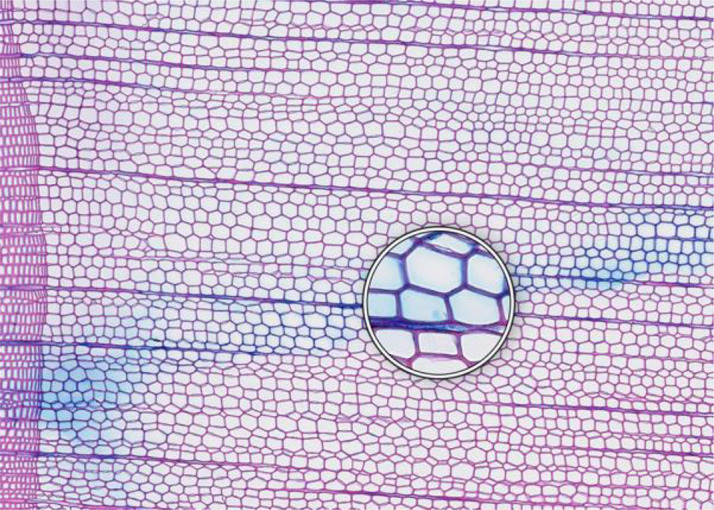 *Picea abies*, Méolans-Revel (France), credit: Lenka Slamova
**Air bubbles**	**DEWAXING, STAINING AND FIXING**
**Description**: The thin section is characterized by the presence of visible air bubbles, which will prevent accurate cell recognition. **Causes**: Air bubbles typically result from improper drying of the section or uneven distribution of mounting medium, such as Euparal, over the tissue. **Prevention**: To minimize air bubble formation, ensure thorough drying of the section before mounting and apply the mounting medium evenly. **Remedy**: Re-staining	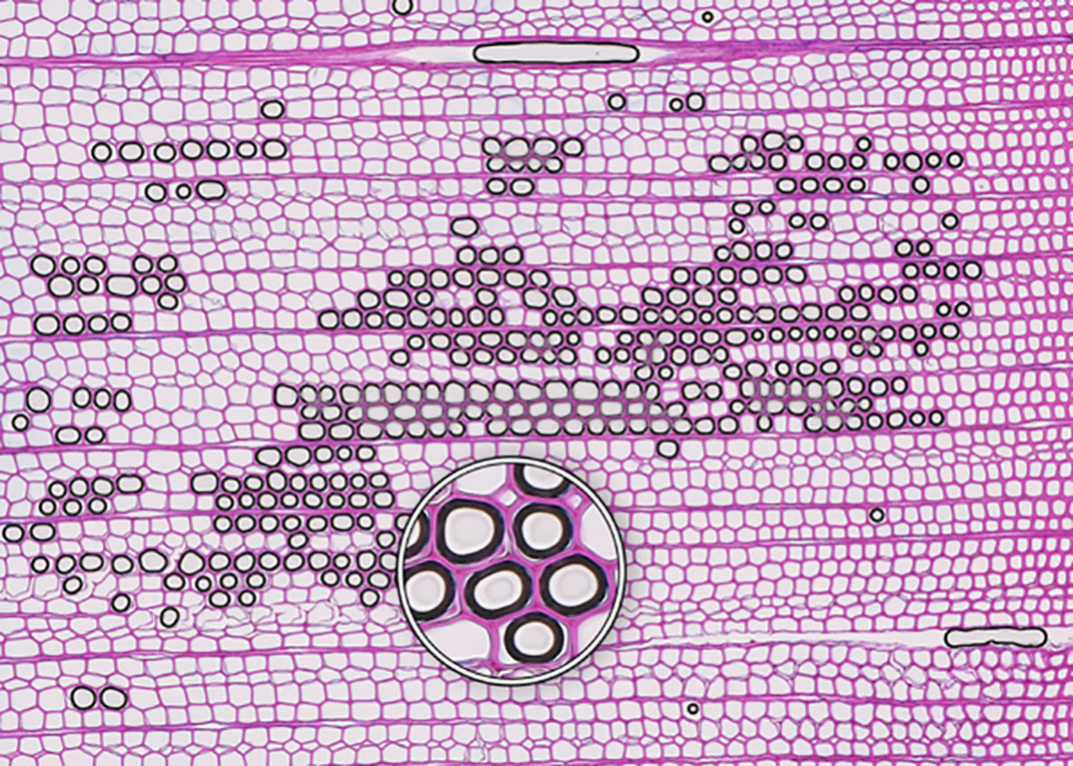 *Picea engelmannii*, T.W. Daniel Experimental Forest, Utah (USA), credit: Matt Bekker
**Faint staining**	**DEWAXING, STAINING AND FIXING**
**Description**: The thin section generally shows faint staining, which reduces the contrast of cellular structures and may distort the recognition of the cells. **Causes**: Faint staining often results from prolonged exposure to ethanol after staining, which can wash out the color from the tissue sections, or insufficient staining time in the staining solution. **Prevention**: Adhere to recommended staining times and avoid excessive rinsing with 96% ethanol. Additionally, ensure that the staining solutions are fresh and properly mixed. **Remedy**: Re-staining	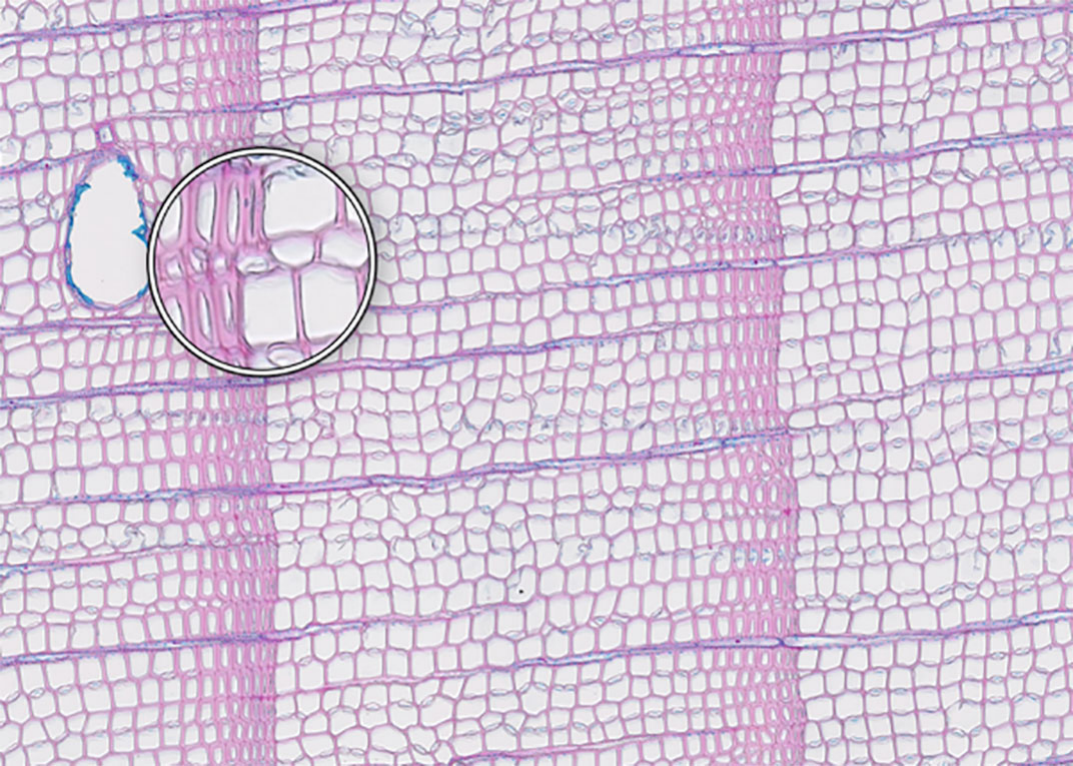 *Picea abies*, Hirschwang (Austria), credit: Lenka Slamova
**Stained lumen**	**DEWAXING, STAINING AND FIXING**
**Description**: The thin section shows staining residue in the lumen, potentially leading to distorted detection of the cellular structures. **Causes**: Insufficient rinsing of the staining solution. **Prevention**: Adhere to the prescribed timing protocols for staining and consider rinsing samples thoroughly after staining to remove excess dye. **Remedy**: Re-staining	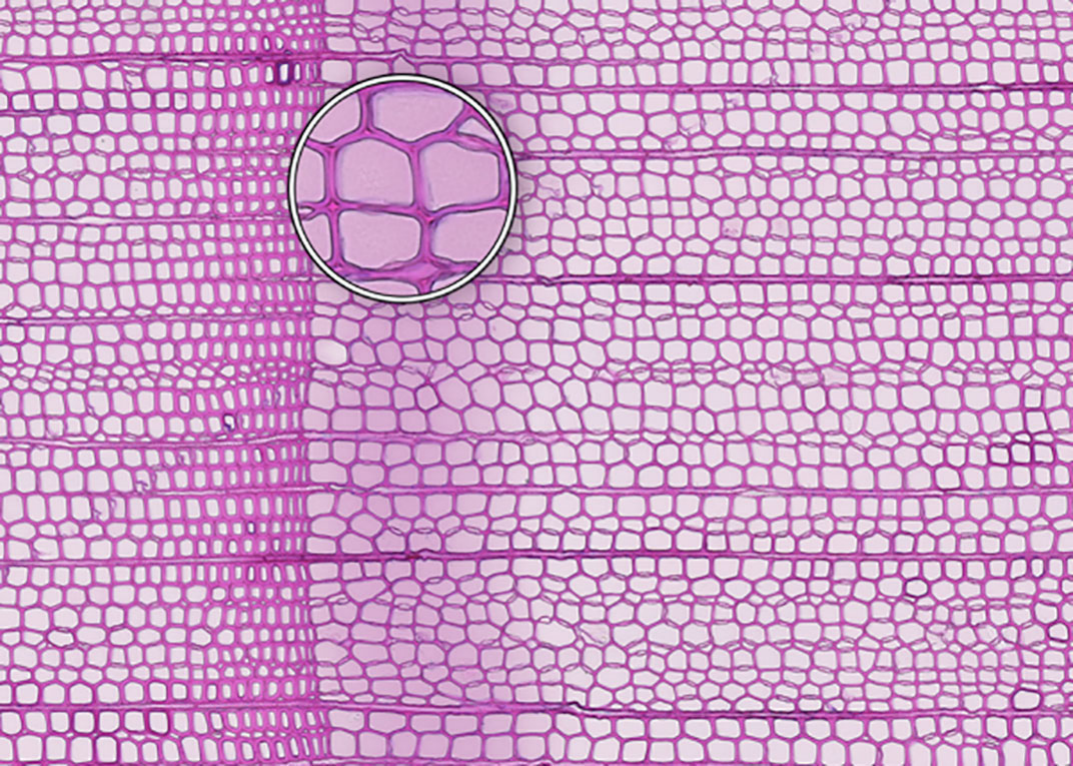 *Picea engelmannii*, T.W. Daniel Experimental Forest, Utah (USA), credit: Matt Bekker
**Paraffin crystals**	**DEWAXING, STAINING AND FIXING**
**Description**: The thin section has paraffin residue, manifesting as a cloud of small bubbles on the tissue that obscure cellular details and can interfere with cell detection and measurements. **Causes**: Paraffin residue typically results from insufficient washing or incomplete removal of paraffin during the dewaxing process. **Prevention**: Adhere to recommended bath duration times. Additionally, consider changing the UltraClear^TM^ solution during the staining process to enhance paraffin removal. **Remedy**: Re-sectioning	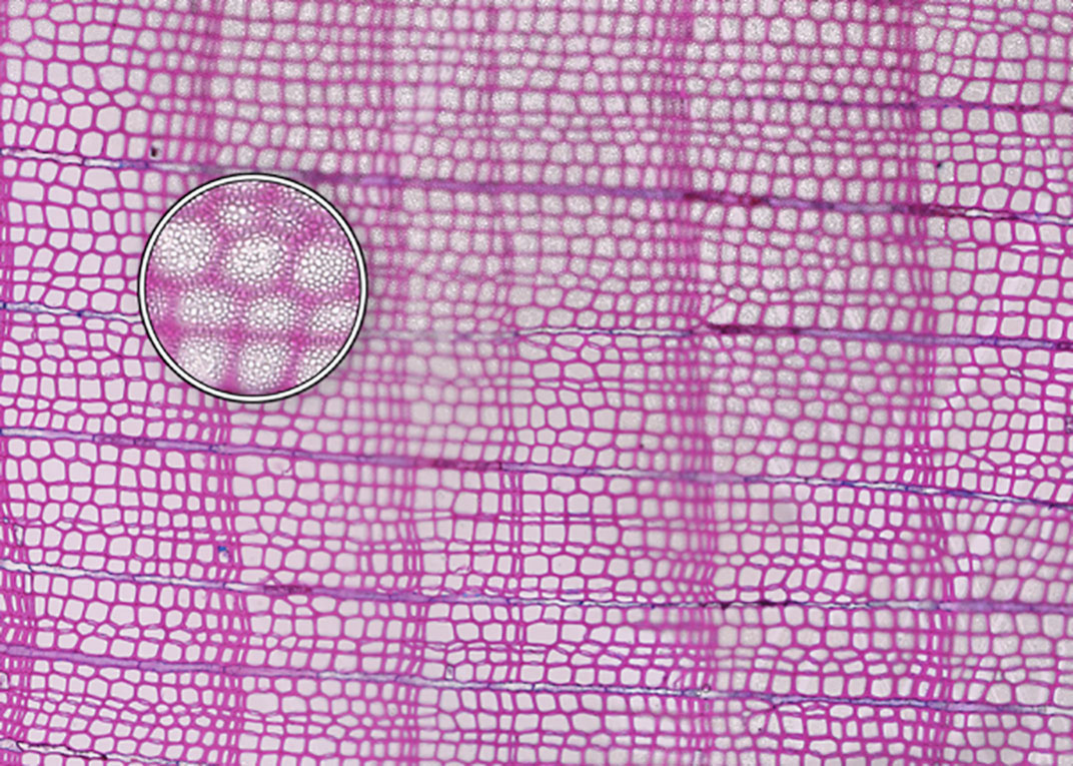 *Austrocedrus chilensis*, Bardas Blancas (Argentina), credit: Marina Fonti
**Blue vails in lumina**	**DEWAXING, STAINING AND FIXING**
**Description**: Some cell lumina in the thin section show a blue veil, characterized by a thin, blueish layer that can prevent the recognition of cell structures. **Causes**: The cause of this issue is not fully clear, it could be torn pit membranes or deposition of substances into the affected lumina. **Prevention**: A brief bleaching treatment (60-90 sec) with a 10 % sodium hypochlorite solution (“Javel water”) between ethanol and staining might help eliminate the blue veils. **Remedy**: Re-sectioning. Alternatively, a brief bleaching treatment between ethanol and staining can help eliminate any unwanted coloration and minimize the risk of a blue veil formation.	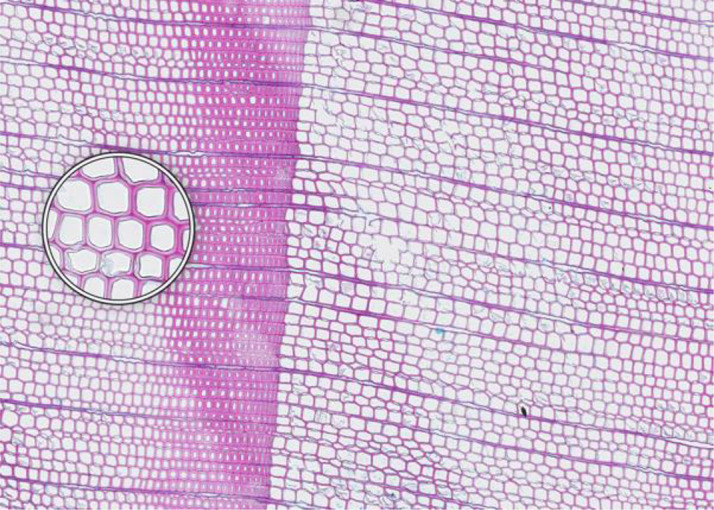 *Pinus sylvestris*, Torneträsk (Sweden), credit: Marina Fonti
**Dirty cover glass**	**IMAGING**
**Description**: The thin section appears smudged or stained, because it is obscured by a dirty cover glass, which may lead to misinterpretation of the cellular structures. **Causes**: This issue is typically caused by inadequate cleaning of the cover glass before imaging, resulting in dust or other contaminants being trapped on the cover glass and/or below the slide. **Proposed Solution**: Ensure thorough cleaning of all cover glass surfaces with a razor blade to remove bulky pollutions such as drops of embedding media and ethanol to remove smaller pollutions. Handle cover glasses by the edges to minimize the risk of making them dirty again. **Remedy**: Re-imaging.	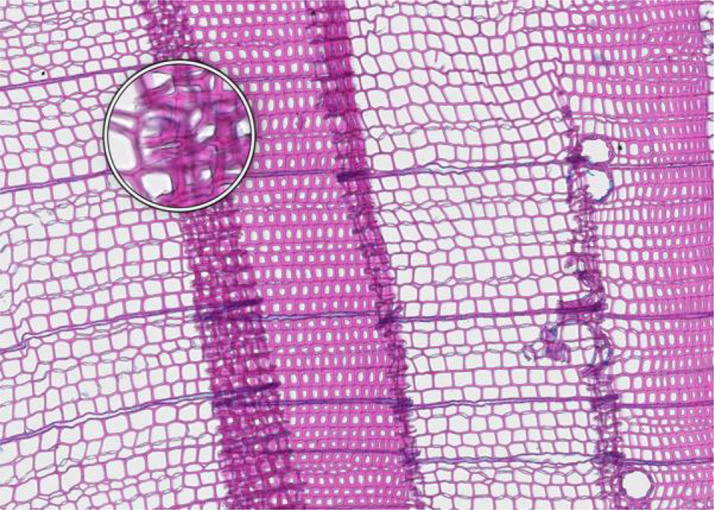 *Cedrus atlantica*, Tazaot (Northern Morocco), credit: Daniel Nievergelt
**Out of focus**	**IMAGING**
**Description**: The thin section appears out of focus, leading to blurry (parts of the) images and difficulty in accurately identifying cellular structures. **Causes**: This issue typically arises from uneven thickness of the section or extensive waviness of the thin section between the slide and the cover glass, which can result in varying focal depths during imaging. **Proposed Solution**: Ensure careful sectioning techniques to maintain consistent thickness and proper positioning of the magnets during hardening of the embedding medium. **Remedy**: Re-imaging with a larger z-stack to better embrace the thin section in all parts, i.e. with a z-position that is in focus	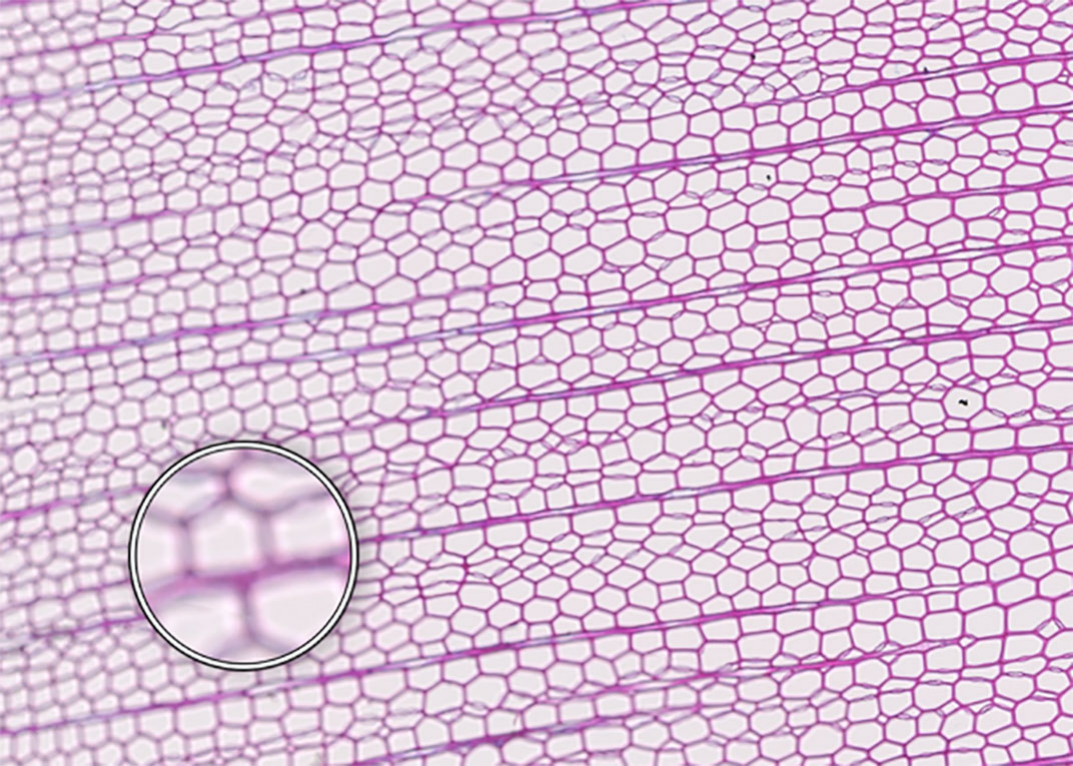 *Picea abies*, Kazitiskis (Lithuania), credit: Lenka Slamova

This table outlines typical issues encountered when preparing thin sections of conifer wood, along with corresponding troubleshooting routines. Re-embedding refers to the process of melting the paraffin block with the wood sample in an embedding station, followed by re-embedding with the correct arrangement of rings and tracheids/vessels, cleaning the cassette, trimming, sectioning, staining, and imaging Re-sectioning involves producing additional thin sections from the same paraffin block, staining, and imaging. Re-staining involves carefully removing the coverslip from the thin section by placing the slide in 96% ethanol for 2 hours. The thin section is then stained again in a Safranin-Astra blue solution, rinsed with water and ethanol, and remounted in Euparal with a new coverslip before imaging. Re-imaging indicates re-capturing the slide with adjusted settings or after removing the issue.

All images shown have the same field of view of 1.985 mm (width) by 1.322 mm (height) and the same orientation (left to right corresponds to pith to bark).

## 4 Equipment and time management

To assist other laboratories interested in adopting and implementing the whole or parts of this protocol, we have included the list of equipment used for this protocol (**Supplementary Table 2**) and a time management plan for a production rate of 100 small or 48 thicker samples per week (**Supplementary Table 3**). These documents are tailored to our lab infrastructure, operators, and material studies, and may require adjustment and improvement based on the specific environment and personnel.

## Data Availability

The original contributions presented in the study are included in the article and in the **Supplementary Material**. Further inquiries can be directed to the corresponding author.

## Footnotes

1
https://www.youtube.com/watch?v=FUddCGVr0bY&t=6s

2
https://www.youtube.com/watch?v=aBC6BR58zT4

3
https://www.youtube.com/watch?v=-hHj-FLfAaQ

4
https://www.youtube.com/watch?v=5FdB9jicvcU

5
https://www.youtube.com/watch?v=ZiDsdT9wfJM
