# Supplementary material for: A protocol for high-quality sectioning for tree-ring anatomy
Source: Front Plant Sci. 2025 Feb 26;16:1505389. doi: 10.3389/fpls.2025.1505389 (PMC11907197; doi:10.3389/fpls.2025.1505389)
Supplement: Supplementary file 1 [file DataSheet1.docx]

**Table 1. Common issues and recommended troubleshooting when preparing thin sections.** This table outlines typical issues encountered when preparing thin sections of conifer wood, along with corresponding troubleshooting routines. **Re-embedding** refers to the process of melting the paraffin block with the wood sample in an embedding station, followed by re-embedding with the correct arrangement of rings and tracheids/vessels, cleaning the cassette, trimming, sectioning, staining, and imaging **Re-sectioning** involves producing additional thin sections from the same paraffin block, staining, and imaging. **Re-staining** involves carefully removing the coverslip from the thin section by placing the slide in 96% ethanol for 2 hours. The thin section is then stained again in a Safranin-Astra blue solution, rinsed with water and ethanol, and remounted in Euparal with a new coverslip before imaging. **Re-imaging** indicates re-capturing the slide with adjusted settings or after removing the issue.

All images shown have the same field of view of 1.985 mm (width) by 1.322 mm (height) and the same orientation (left to right corresponds to pith to bark).

| **Microcracks** | **SAMPLE COLLECTION AND PREPARATION** | |  |
| --- | --- | --- | --- |
|  |  | |  |
| **Description**: Small fissures typically running parallel or oblique to the growth rings of the wood. These cracks typically leave cells intact, but affect cell position and don’t allow wall thickness measurements adjacent to the cracks.  **Causes**: Microcracks often result from mechanical stress during manual coring. This can occur due to unstable borer guidance when inserting the borer into the trunk or from using a dull borer.  **Prevention**: Use a sharp borer, ideally powered by an electric drill, to ensure smooth and consistent friction. Employing a 10 mm thick borer can reduce the likelihood of microcrack formation, as it provides more core stability during collection and allowing for the collection of wood sections further from the core surface, which are less likely to be affected by cracks.  **Remedy**: None; use affected sample for measurements with caution. | 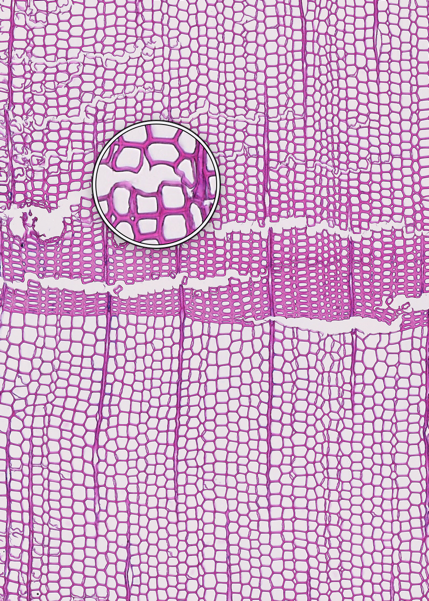*Picea engelmannii*, T.W. Daniel Experimental Forest, Utah (USA), credit: Matt Bekker | |  |
|  |  | |  |
|  |  | |  |
| **Microorganisms** | **SAMPLE COLLECTION AND PREPARATION** | |  |
|  |  | |  |
| **Description**: Thin section characterized by the presence of microorganisms, such as fungi or bacteria, within the wood tissue. These organisms may appear as dark spots, irregular structures, or hyphal networks, often disrupting the normal cell structure and resulting in less intense staining in the affected areas. This disruption can complicate the identification of the cells.  **Causes**: Microbial infiltration typically results from wood decay or contamination, often occurring in compromised or decaying wood.  **Prevention**: Collect samples from healthy wood and ensure they are stored under dry conditions.  **Remedy**: None; heavily affected samples should be excluded from analysis, as they may yield unreliable data. | 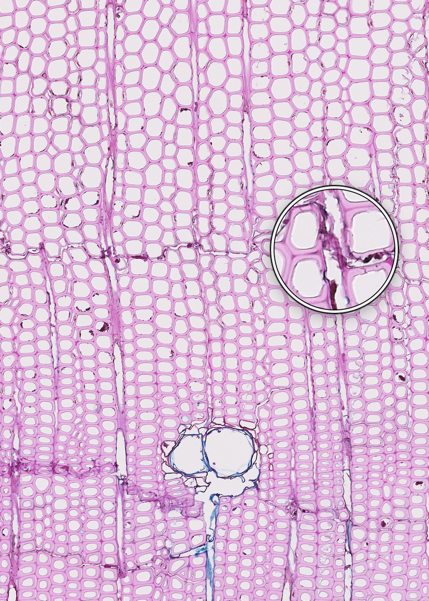  *Pinus pinea*, Central Spain, credit: Macarena Ferriz Nuñez | |  |
|  |  | |  |
| **Cell walls broken in random directions** | | **SAMPLE COLLECTION AND PREPARATION** | |
|  |  | |  |
| **Description**: Thin section with tracheid walls broken or disrupted in different directions. This damage prevents the correct recognition of the cells.  **Causes**: Broken cell walls in all directions often result from an inadequate surface preparation, particularly sanding. This improper preparation can obstruct effective paraffin infiltration, leaving visible signs of sanding and contributing to structural weakness during sample sectioning.  **Prevention**: Use a fine-toothed saw for extracting radial bars from larger wood pieces like stem discs. Smooth and level the surface of increment cores and radial bars with a core microtome (instead of sanding) to enhance paraffin infiltration. Remove enough surface wood with a rotary microtome to avoid damaged cells before thin sectioning. Ensure thorough paraffin infiltration by vacuuming the sample immersed in water beforehand.  **Remedy**: None; utilize an area of exclusion if the affected region is relatively small. | 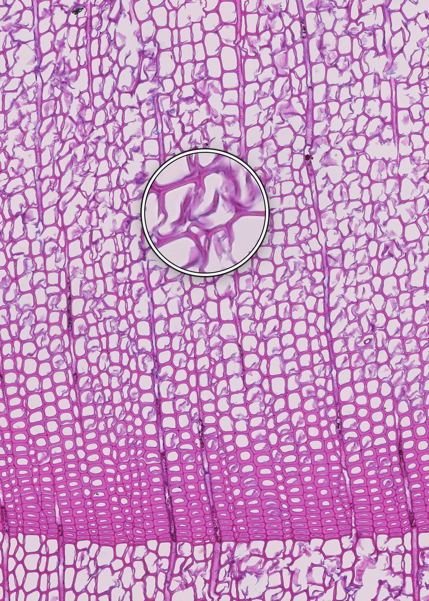  *Abies marocana*, Tazaot (Northern Morocco), credit: Patrick Fonti | |  |
| **Red lumen** | **SAMPLE COLLECTION AND PREPARATION** | |  |
|  |  | |  |
| **Description**: The thin section shows lumina that are partly filled with red-stained inclusions that can prevent the recognition of cell structures.  **Causes**: This issue is likely caused by the deposition of gums or other substances into the affected lumina.  **Prevention**: Such depositions may be species-specific and often don’t disappear even after Soxhlet extraction. There might be wood samples that are less affected.  **Remedy**: None; use affected sample for measurements with caution. | 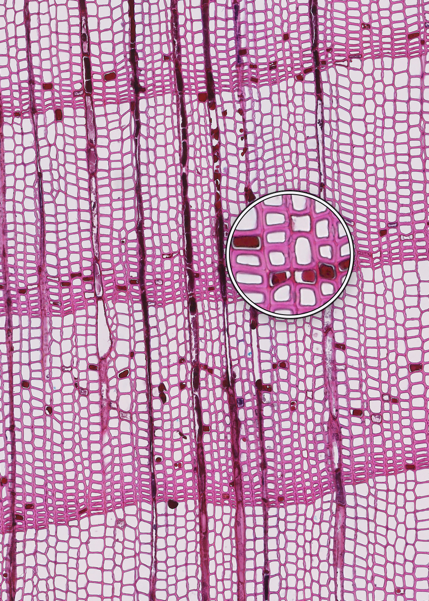  *Fitzroya cupressoides*, Rio Alerce (Argentina), credit: Marina Fonti | |  |
|  |  | |  |
|  |  | |  |
| **Compression wood** | **SAMPLE COLLECTION AND PREPARATION** | |  |
|  |  | |  |
| **Description**: The thin section shows compression wood, characterized by abnormal cell structures and density changes that can affect the overall analysis of the wood sample. This issue is different from all above as there might be no technical problem, but rather an unfortunate choice of wood material.  **Causes**: Compression wood typically develops in response to mechanical stress or gravitational forces, leading to altered growth patterns and irregular cell wall formation.  **Prevention**: Select compression wood free areas or samples from stable and healthy trees.  **Remedy**: None; don’t use affected samples or tree rings for measurements, unless these structures are the target of the investigation. | 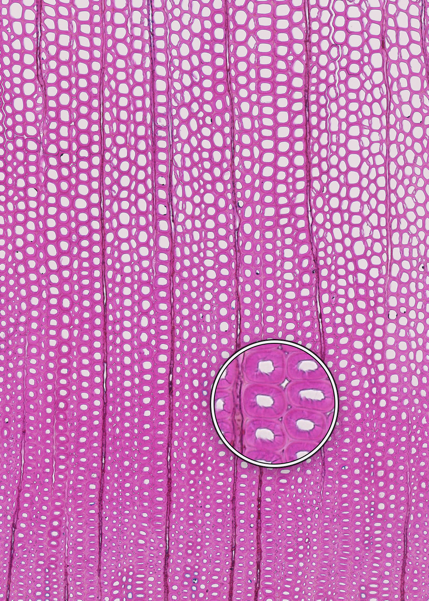  *Larix sibirica*, Yamal (Siberia), credit: Marina Fonti | |  |
|  |  | |  |
| **Cell walls directionally disrupted** | **PARAFFIN INFILTRATION AND EMBEDDING** | |  |
|  |  | |  |
| **Description**: The thin section is characterized by disruptions in the cell wall that all point in the same direction, which can hinder cell measurement.  **Causes**: This type of broken cell wall often results from insufficient paraffin infiltration during embedding. This insufficient infiltration can occur if the sample surface has not been properly prepared or if the sample has not been adequately vacuumed, leaving weak spots that are susceptible to fracturing when the microtome blade strikes the tracheids.  **Prevention**: Section the wood surface to enhance paraffin infiltration, ensuring that the sections are deep enough. Additionally, vacuum the sample immersed in water before paraffin infiltration to promote thorough infiltration.  **Remedy**: None; utilize an area of exclusion if the affected region is relatively small. | 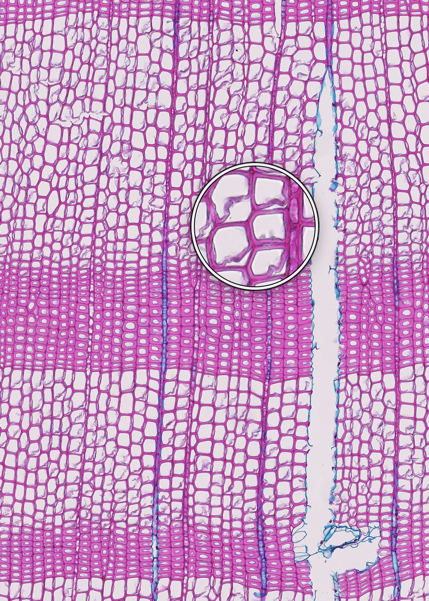  *Pinus sylvestris*, Gotland (Sweden), credit: Marina Fonti | |  |
|  |  | |  |
| **Fuzzy lumen outlines** | **PARAFFIN INFILTRATION AND EMBEDDING** | |  |
|  |  | |  |
| **Description**: The thin section shows unsharp cell lumina, indicating that tracheid orientation was not orthogonal during sectioning, leading to a distorted appearance and underestimated lumen area. Tangential misalignment causes pits to appear in a side view, while radial misalignment makes rays appear shortened across the section.  **Causes**: Fuzzy lumina typically arise from improper sample positioning during the embedding process.  **Prevention**: Ensure correct sample orientation during both sample collection and embedding to avoid misalignment.  **Remedy**: Re-embedding | 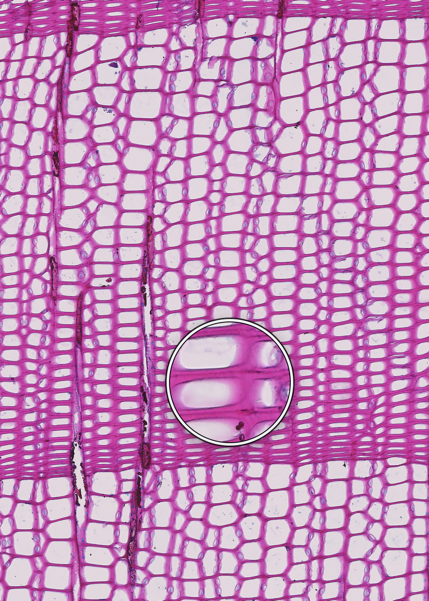  *Cedrus atlantica*, Tazaot (Northern Morocco), credit: Daniel Nievergelt | |  |
|  |  | |  |
| **Cell walls broken along lines** | **SECTIONING AND FLOATATION** | |  |
|  |  | |  |
| **Description**: The thin section is characterized by the presence of broken tracheids and cracks along lines. This prevents accurate measurement of the disrupted cells.  **Causes**: These breaks often result from using a blunt or damaged microtome blade during sectioning. Additionally, hard particles within the sample that are pushed through the sample during sectioning can result in similar damage.  **Prevention**: Use a sharp blade for sectioning to ensure even sections.  **Remedy**: None; utilize an area of exclusion if the affected region is relatively small. | 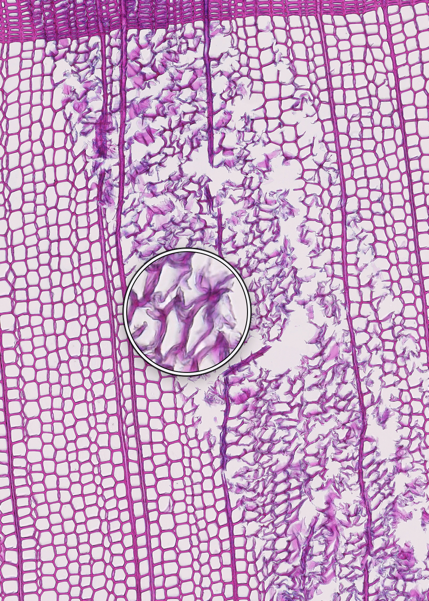  *Picea engelmannii*, T.W. Daniel Experimental Forest, Utah (USA), credit: Matt Bekker | |  |
|  |  | |  |
|  |  | |  |
| **Light streaks** | **SECTIONING AND FLOATATION** | |  |
|  |  | |  |
| **Description**: Light streaks are characterized by uneven sections in the wood sample, leading to varying tissue thickness or minor cell wall disruption. This can introduce biases in measurements, affecting the accuracy of the data.  **Causes**: This issue is typically caused by an improperly fixed microtome blade or poorly fixed samples, causing the blade to lose contact with the sample during sectioning. Additionally, cracked paraffin blocks can contribute to this issue.  **Prevention**: Ensure the microtome blade is sharp and securely fixed, that the sample is properly clamped, and that the paraffin block is intact.  **Remedy**: None. | 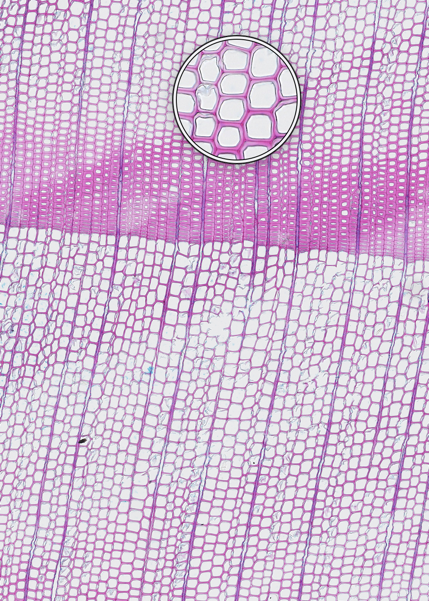  *Picea abies*, Croda da Lago (Italy), credit: Lenka Slamova. | |  |
|  |  | |  |
| **Folded tissue** | **SECTIONING AND FLOATATION** | |  |
|  |  | |  |
| **Description**: The thin section is characterized by overlaying tissues, typically occurring at the transition between earlywood and latewood. This overlap prevents correct cell recognition and thus results in locally wrong measurements.  **Causes**: Overlaying tissues often result from density differences between earlywood and latewood, causing instability during sectioning. Improper tape sliding or handling in the water bath can also lead to tissue folding.  **Prevention**: Reversing the sample orientation in the microtome clamp can reduce pressure from density differences. Proper tape sliding and careful handling in the water bath further minimize folding.  **Remedy**: None; utilize an area of exclusion if the affected region is relatively small. | 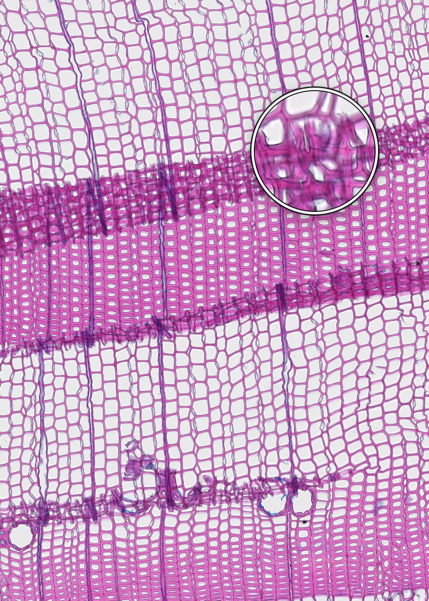  *Picea abies*, Kazitiskis (Lithuania), credit: Lenka Slamova | |  |
|  |  | |  |
| **Overlying tissue fragments** | **SECTIONING AND FLOATATION** | |  |
|  |  | |  |
| **Description**: The thin section shows overlying tissue fragments, where one tissue layer obscures underlying structures, preventing correct cell recognition and resulting in locally wrong measurements.  **Causes**: Overlying tissue fragments often results from contamination due to a dirty microtome sliding table or water bath during sectioning.  **Prevention**: Ensure cleanness of both the microtome and water bath to maintain optimal sectioning conditions.  **Remedy**: None; utilize an area of exclusion if the affected region is relatively small. | 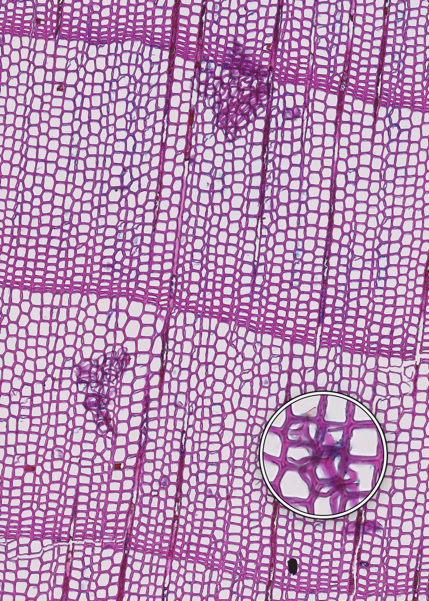  *Larix sibirica*, Yamal (Siberia), credit: Marina Fonti | |  |
|  |  | |  |
| **Albumin residue** | **DEWAXING, STAINING AND FIXING** | |  |
|  |  | |  |
| **Description**: The thin section shows the presence of albumin residue, appearing as a bluish smeared layer behind the tissue. If it is too dark, it may impede correct cell detection.  **Causes**: Albumin residue typically results from an excessive layer of albumin on the slide, leaving protein traces on the wood surface.  **Prevention**: Ensure that the amount of albumin applied to the slide is appropriate and evenly distributed.  **Remedy**: None; use affected sample for measurements with caution. | 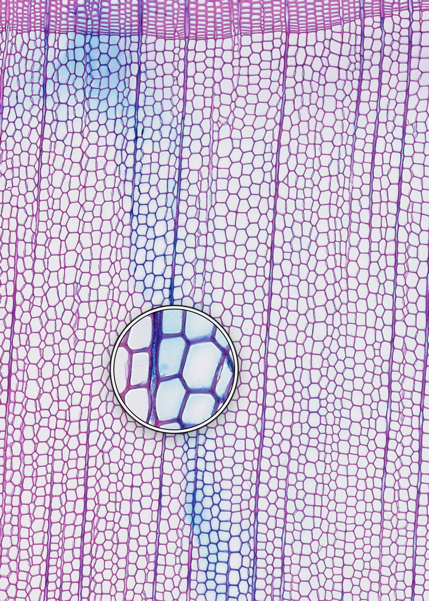  *Picea abies*, Méolans-Revel (France), credit: Lenka Slamova | |  |
|  |  | |  |
|  |  | |  |
| **Air bubbles** | **DEWAXING, STAINING AND FIXING** | |  |
|  |  | |  |
| **Description**: The thin section is characterized by the presence of visible air bubbles, which will prevent accurate cell recognition.  **Causes**: Air bubbles typically result from improper drying of the section or uneven distribution of mounting medium, such as Euparal, over the tissue.  **Prevention**: To minimize air bubble formation, ensure thorough drying of the section before mounting and apply the mounting medium evenly.  **Remedy**: Re-staining | 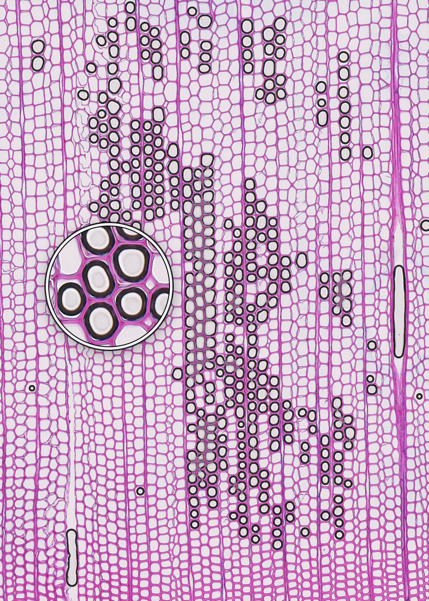  *Picea engelmannii*, T.W. Daniel Experimental Forest, Utah (USA), credit: Matt Bekker | |  |
|  |  | |  |
|  |  | |  |
| **Faint staining** | **DEWAXING, STAINING AND FIXING** | |  |
|  |  | |  |
| **Description**: The thin section generally shows faint staining, which reduces the contrast of cellular structures and may distort the recognition of the cells.  **Causes**: Faint staining often results from prolonged exposure to ethanol after staining, which can wash out the color from the tissue sections, or insufficient staining time in the staining solution.  **Prevention**: Adhere to recommended staining times and avoid excessive rinsing with 96% ethanol. Additionally, ensure that the staining solutions are fresh and properly mixed.  **Remedy**: Re-staining | 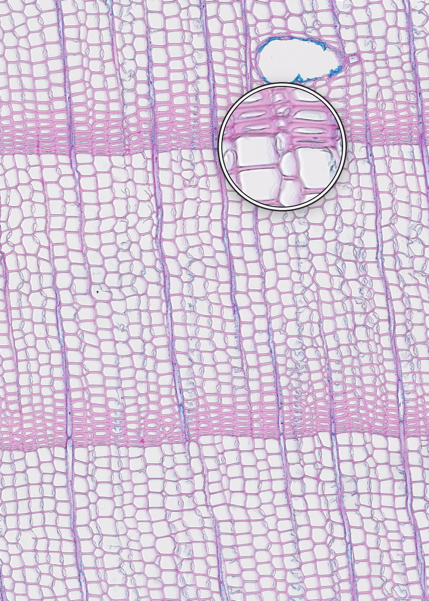  *Picea abies*, Hirschwang (Austria), credit: Lenka Slamova | |  |
|  |  | |  |
|  |  | |  |
| **Stained lumen** | **DEWAXING, STAINING AND FIXING** | |  |
|  |  | |  |
| **Description**: The thin section shows staining residue in the lumen, potentially leading to distorted detection of the cellular structures.  **Causes**: Insufficient rinsing of the staining solution.  **Prevention**: Adhere to the prescribed timing protocols for staining and consider rinsing samples thoroughly after staining to remove excess dye.  **Remedy**: Re-staining | 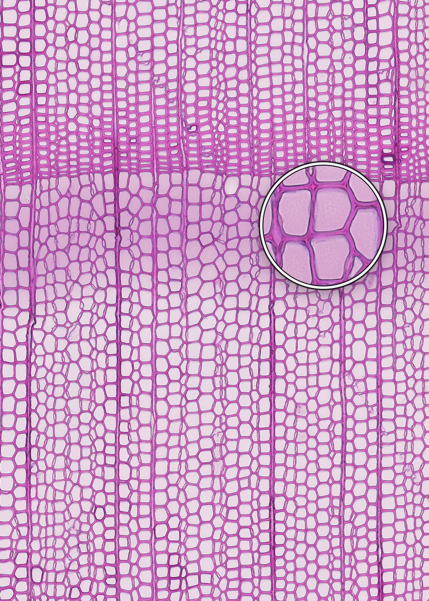  *Picea engelmannii*, T.W. Daniel Experimental Forest, Utah (USA), credit: Matt Bekker | |  |
|  |  | |  |
| **Paraffin crystals** | **DEWAXING, STAINING AND FIXING** | |  |
|  |  | |  |
| **Description**: The thin section has paraffin residue, manifesting as a cloud of small bubbles on the tissue that obscure cellular details and can interfere with cell detection and measurements.  **Causes**: Paraffin residue typically results from insufficient washing or incomplete removal of paraffin during the dewaxing process.  **Prevention**: Adhere to recommended bath duration times. Additionally, consider changing the UltraClear ^TM^ solution during the staining process to enhance paraffin removal.  **Remedy**: Re-sectioning | 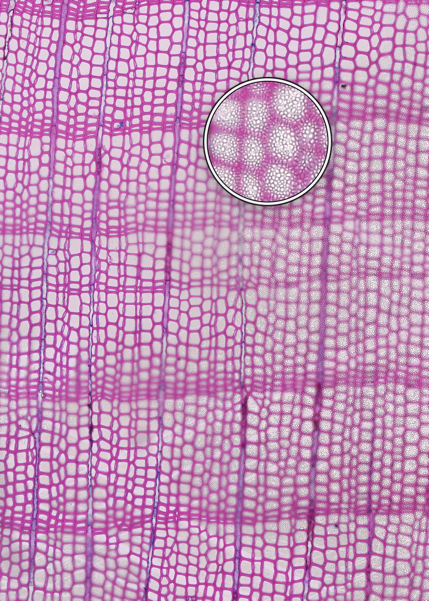  *Austrocedrus chilensis*, Bardas Blancas (Argentina), credit: Marina Fonti | |  |
|  |  | |  |
|  |  | |  |
| **Blue vails in lumina** | **DEWAXING, STAINING AND FIXING** | |  |
|  |  | |  |
| **Description**: Some cell lumina in the thin section show a blue veil, characterized by a thin, blueish layer that can prevent the recognition of cell structures.  **Causes**: The cause of this issue is not fully clear, it could be torn pit membranes or deposition of substances into the affected lumina.  **Prevention**: A brief bleaching treatment (60-90 sec) with a 10 % sodium hypochlorite solution (“Javel water”) between ethanol and staining might help eliminate the blue veils.  **Remedy**: Re-sectioning. Alternatively, a brief bleaching treatment between ethanol and staining can help eliminate any unwanted coloration and minimize the risk of a blue veil formation. | 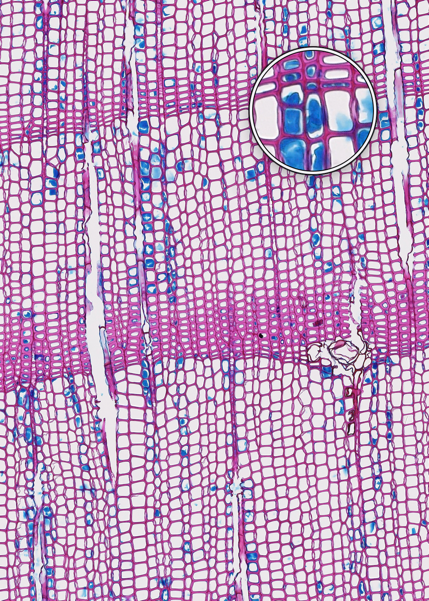  *Pinus sylvestris*, Torneträsk (Sweden), credit: Marina Fonti | |  |
|  |  | |  |
|  |  | |  |
| **Dirty cover glass** | **IMAGING** | |  |
|  |  | |  |
| **Description**: The thin section appears smudged or stained, because it is obscured by a dirty cover glass, which may lead to misinterpretation of the cellular structures.  **Causes**: This issue is typically caused by inadequate cleaning of the cover glass before imaging, resulting in dust or other contaminants being trapped on the cover glass and/or below the slide.  **Proposed Solution**: Ensure thorough cleaning of all cover glass surfaces with a razor blade to remove bulky pollutions such as drops of embedding media and ethanol to remove smaller pollutions. Handle cover glasses by the edges to minimize the risk of making them dirty again.  **Remedy**: Re-imaging. | 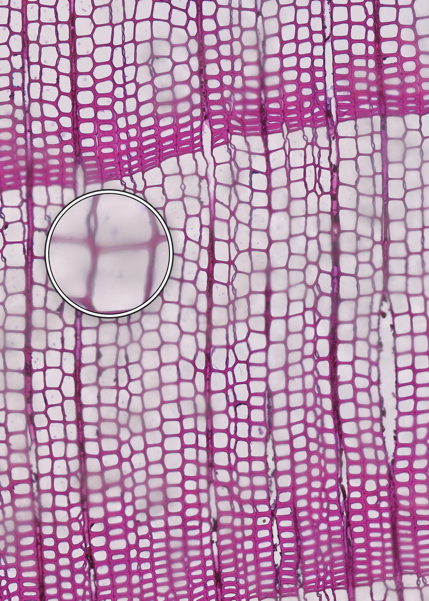  *Cedrus atlantica*, Tazaot (Northern Morocco), credit: Daniel Nievergelt | |  |
|  |  | |  |
|  |  | |  |
| **Out of focus** | **IMAGING** | |  |
|  |  | |  |
| **Description**: The thin section appears out of focus, leading to blurry (parts of the) images and difficulty in accurately identifying cellular structures.  **Causes**: This issue typically arises from uneven thickness of the section or extensive waviness of the thin section between the slide and the cover glass, which can result in varying focal depths during imaging.  **Proposed Solution**: Ensure careful sectioning techniques to maintain consistent thickness and proper positioning of the magnets during hardening of the embedding medium.  **Remedy**: Re-imaging with a larger z-stack to better embrace the thin section in all parts, i.e. with a z-position that is in focus | 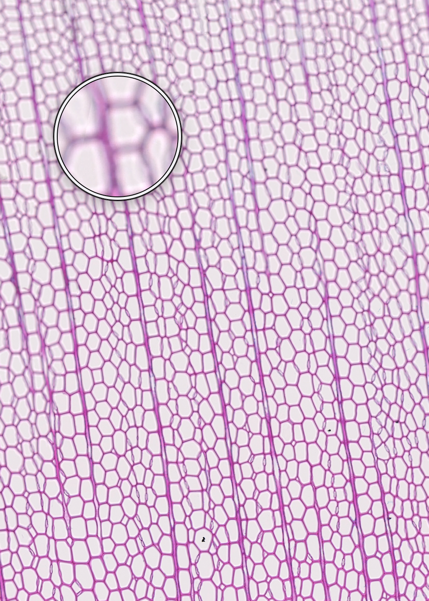  *Picea abies*, Kazitiskis (Lithuania), credit: Lenka Slamova | |  |
|  |  | |  |
