# Supplementary material for: A protocol for high-quality sectioning for tree-ring anatomy
Source: Front Plant Sci. 2025 Feb 26;16:1505389. doi: 10.3389/fpls.2025.1505389 (PMC11907197; doi:10.3389/fpls.2025.1505389)
Supplement: Supplementary file 2 [file Table2.docx]

**Supplementary Table 2**. **Equipment list**

| **Processing stage** | **Type** | **Supplier** | **Art. Nr** | **Description** | | | **Comments** | | |  |  |  |  |
| --- | --- | --- | --- | --- | --- | --- | --- | --- | --- | --- | --- | --- | --- |
| **1. Sample collection and preparation** | |  |  |  | | |  | | |  |  |  |  |
| Water-resistant soft pencil | C | Galaxus | 12223367 | Stabilo aquarellable All 8008 | | |  | | |  |  |  |  |
| Increment borer | D | Forestry Suppliers | 63251 | 3-Thread Increment Borers Haglöf | | |  | | |  |  |  |  |
| Drill and Torque setting adapter | D | Metabo | 602359660 and 627256000 | Cordless drill / screwdriver BS 18 LTX BL Q I (602359660) with PowerX3 torque setting adapter (627256000) | | | |  | | | | | |
| WSL Core adapter | D | WSL |  | Core adapter | | |  | | |  |  |  |  |
| Whatman paper or cardboard | C | Office supplies |  | Paper and Cardboard for storage and sample stabilisation | | |  | | |  |  |  |  |
| Silicone glue | C | OBI | 3123387 | Transparent and neutral constriction silicon gel (not acrylic or silicone/acrylic mix and not acidic) | | | |  | |  |  |  |  |
| Band saw | D | Felder group | 500-13-351 | Band saw | | | e.g.; Hammer Bandsäge N2-35 | | |  |  |  |  |
| Fine bandsaw blade | C | Felder group | 13.07.3506 | Band saw blade | | | e.g.; FLEX-BACK-Sägeblatt with 0.6 mm teeth | | |  |  |  |  |
| WSL core microtome | D | WSL |  | Core Microtome | | |  | | |  |  |  |  |
| Disposable blades | C | Office supplies | BA-170 | Light-Duty A type NT Cutter blades (black and ultrasharp included) | | |  | | |  |  |  |  |
| **2. Removal of extractants, splitting, labeling, and orienting** | | | | |  | | | |  | |  |  |  |
| Soxhlet apparatus with extractor, Dimroth cooler, Roundflask and Extraction thimbles | L | Roth | Y356.1, AA60.1, ACK0.1 and L867.1 | Soxlet extractor, Dimroth cooler, Roundflask, and Extraction thimbles | | |  | | |  | | |  |
| Electric heater for Soxhlet apparatus | D | VWR | ELET36002-18 | Electric heater | | |  | | |  | | |  |
| Ethanol 96% | C | WSL internal /Alcosuisse |  | Ethanol Tech. 96% | | |  | | |  | | |  |
| Water-resistant soft pencil | C | Galaxus | 12223367 | Stabilo aquarellable All 8008 | | |  | | |  | | |  |
| Sharp cutter knife | L | Office supplies | various | Light-Duty A type NT Cutter (various models) | | | e.g.; NT CUTTER A-300RP, Artikelnummer 184 301 , buehlerzuerich.ch | | |  | | |  |
| Hammer | L | OBI | 1224237 | LUX Holzhammer Comfort 70 mm | | |  | | |  | | |  |
| Embedding cassettes (5 mm and 10 mm height) | C | VWR | 720-0887 and 720-1627 | Simport Histocette M490 and Simport Macrocette M512 | | | various colors available article Nr. for white version | | |  | | |  |
| **3. Paraffin infiltration and embedding** | |  |  |  | | |  | | |  | | |  |
| Tissue processor | D | Biosystems | 14042230535 | Leica TP 1020, Biosystems, Nussloch, Germany | | |  | | |  | | |  |
| Ethanol 75% | C | WSL internal /Alcosuisse |  | Ethanol 75% diluted from Ethanol Tech. 96% | | |  | | |  | | |  |
| Ethanol 96% | C | WSL internal /Alcosuisse |  | Ethanol Tech. 96% | | |  | | |  | | |  |
| Ethanol 100% | C | Biosystems | 84-3110-00 | Ethanol Asbolut with 2% MEK | | |  | | |  | | |  |
| Ultraclear | C | Biosystems | 3905.9010PE | UltraClear™ (xylol substitute) | | |  | | |  | | |  |
| Paraffin | C | Biosystems | 81-0763-00 | Paraplast™ (melting point 56°C) | | |  | | |  | | |  |
| Vacuum chamber and pump | D | Shenzhen Haocheng Instrument Co., Ltd | | e.g.; 2 Gal (8L) Vacuum Chamber Kit with 2.5CFM (1.4L/s) 220V Vacuum Pump,25cm*20cm Stainless Steel Degassing Chamber | | | |  | |  | | |  |
| Embedding station | D | Biosystems | 14039357257 | Leica HistoCore Arcadia H | | | Also includes a cooling plate | | |  | | |  |
| Molds | L | Biosystems | 81-0264-00 and 47-2005-00 | 37 x 24 x 09 mm and 37 x 24 x 05 mm | | | for 1 cm big /deep sample and 5mm cores | | |  | | |  |
| Straight pointed tweezers | L | Roth | 2802.1 | Tweezers straight pointed, 130 mm | | | up to personal preference many potential sources sizes and shapes. Tweezers for ESP work particulary well. | | |  | | |  |
| Cooling plate | D | Biosystems | 14039357262 | Leica HistoCore Arcadia C | | | Together with the embedding station | | |  | | |  |
| **4. Trimming** |  |  |  |  | | |  | | |  | | |  |
| Sharp cutter knife | L | Office supplies | iA-120P | Light-Duty A type NT Cutter (various models) | | | e.g.; NT CUTTER A-300RP, Artikelnummer 184 301 , buehlerzuerich.ch | | |  | | |  |
| Rotary microtome | D | Biosystems | 14051956472 | HistoCore AUTOCUT | | |  | | |  | | |  |
| High-profile disposable blades | C | Biosystems | 81-0393-00 | Micros HP | | |  | | |  | | |  |
| Low-profile disposable blades | C | Biosystems | 81-0353-00 | FEATHER® N35 | | |  | | |  | | |  |
| Dissection needle | L | Roth | KX93.1 | Dissecting needles Plastic handle, straight | | |  | | |  | | |  |
| Brush | L | DIY / Hobby and art store | various | Pelican Hair brushes no. 23 (size 2-3 or as preferred) | | |  | | |  | | |  |
| **5. Sectioning and floatation** |  |  |  |  | | |  | | |  | | |  |
| Rotary microtome | D | Biosystems | 14051956472 | HistoCore AUTOCUT | | |  | | |  | | |  |
| High-profile disposable blades | C | Biosystems | 81-0393-00 | Micros HP | | |  | | |  | | |  |
| Lw-profile disposable blades | C | Biosystems | 81-0353-00 | FEATHER® N35 | | |  | | |  | | |  |
| Brush | L | DIY / Hobby and art store | various | Pelican Hair brushes no. 23 (size 2-3 or as preferred) | | |  | | |  | | |  |
| Pointed-tipped tweezers | L | Roth | KX93.1 | Dissecting needles Plastic handle, straight | | |  | | |  | | |  |
| Water bath | D | VWR | 720-3029 | VWR® W20, Histology Water Bath | | | combined drying surface as well as water bath | | |  | | |  |
| Albumin | C | Roth | P049.1 | Protein glycerol | | |  | | |  | | |  |
| Microslides | C | Biosystems | 85-0711-00 | SuperFrost® slides with 90° ground edges | | | ground edge is a convenience to avoid cutting injuries. labeling surface is recommended | | |  | | |  |
| Label printer with software | D | Zebra | 16463848 | Zebra ZD621 with ZebraDesigner Professional 3 | | | we use the GX430t which was discontinued in 2022 the ZD621 is the successor. The software is a free version that does not allow to read lists from excel sheets | | |  | | |  |
| Chemical-resistant printed sticker | C | Labid-technologies | N0FTT-149C1-2WH | Xylene And Solvent Resistant Labels 25.4 x 7mm | | | also available for cassettes (untested) | | |  | | |  |
| Slide racks | L | VWR | 631-9321 with 631-9329 and 631-9328 | Glass rack for 10 slides with metal handle for glass rack and staining jar with lid | | | |  | |  | | |  |
| Embedding carton box | L | Histocom | 40109 | Diastore Block 12 | | |  | | |  | | |  |
| **6. Dewaxing, staining, and fixing** | |  |  |  | | |  | | |  | | |  |
| Slide racks | L | VWR | 631-9321 with 631-9329 and 631-9328 | Glass rack for 10 slides with metal handle for glass rack and staining jar with lid | |  | | | |  | | |  |
| Oven | D | Faust | 4658060 | BINDER FD 115 Drying and heating chambers with forced convection | | |  | | |  | | |  |
| Safranin | C | Roth | T129.3 | Safranin O (C.I. 50240) | | |  | | |  | | |  |
| Astra blue | C | Roth | 5165.2 | Astra blue FM (C.I. 48048) | | |  | | |  | | |  |
| Xylene | C | Biosystems | 84-2510-00 | Xylol | | |  | | |  | | |  |
| Ethanol 96% | C | WSL internal /Alcosuisse |  | Ethanol Tech. 96% | | |  | | |  | | |  |
| Euparal | C | Roth | 7356.1 | Euparal | | |  | | |  | | |  |
| Cover glass 50 mm | C | VWR | 631-1574 | Cover Slips | | | various sizes available article Nr. is for 24×50 mm | | |  | | |  |
| Pipette with bulb | C | Roth | 4518.1 with 8404.1 | Pasteur pipettes without cotton plug with Pipette bulb nature | | |  | | |  | | |  |
| Iron plate | L | DIY / home improvement store | various | Ferrous metal plate | | | size depending on your oven shape. recommended strength 2mm | | |  | | |  |
| Magnets | L | Kaiserkraft | 177403 49 | Rectangular office magnets 22 x 55 x 8 mm | | |  | | |  | | |  |
| Magnets | L | Supermagnete | FE-S-20-05 | Ferrite magnets Y35 20mm diameter 5mm height | | |  | | |  | | |  |
| Layer of polyethylene | C | Elke-Plastic | 1SF200100 | LDPE Tubular film 200mm diameter 100µm thickness | | |  | | |  | | |  |
| Commercial razorblades | C | Biosystems | 71970 | Single Edge Stainless Steel Teflon Coated Blades | | | plain uncoated razorblades work as well | | |  | | |  |
| Microslide boxes | L | VWR | 631-1504 | Slide Boxes with Lid | | | various colors available. Article Nr. for white version | | |  | | |  |
| **7. Imaging** |  |  |  |  | | |  | | |  | | |  |
| Slide scanner | D | Zeiss |  | Zeiss Axio Scan.Z1 slide scanner, Carl Zeiss, Germany | | |  | | |  | | |  |

**Suppliers**: Alcosuisse ([www.alcosuisse.ch](http://www.alcosuisse.ch)); Biosystems ([www.biosystems.ch](http://www.biosystems.ch)); Elke-Plastic ([www.elke-plastic.ch](http://www.elke-plastic.ch)); Faust ([www.faust.ch](http://www.faust.ch)); Felder Group ([www.felder-group.com](http://www.felder-group.com)); Forestry Suppliers ([www.forestry-suppliers.com](http://www.forestry-suppliers.com)); Galaxus ([www.galaxus.ch](http://www.galaxus.ch)); Histocom ([www.histocom.ch](http://www.histocom.ch)); Kaiserkraft ([www.kaiserkraft.ch](http://www.kaiserkraft.ch)); Labid-technologies ([www.labidt.eu](http://www.labidt.eu)); Metabo ([www.metabo.ch](http://www.metabo.ch)); OBI( [www.obi.ch](http://www.obi.ch)); Roth ([www.carlroth.com/ch](http://www.carlroth.com/ch)); Shenzhen Haocheng Instrument ([www.hcinstrument.cn](http://www.hcinstrument.cn)); Supermagnete ([www.supermagnete.ch](http://www.supermagnete.ch)); VWR / Avantor ([www.ch.vwr.com/store](http://www.ch.vwr.com/store)); Zebra ([www.zebra.com](http://www.zebra.com))
