# Supplementary material for: A protocol for high-quality sectioning for tree-ring anatomy
Source: Front Plant Sci. 2025 Feb 26;16:1505389. doi: 10.3389/fpls.2025.1505389 (PMC11907197; doi:10.3389/fpls.2025.1505389)
Supplement: Supplementary file 3 [file Table3.docx]

**Supplementary Table 3**. The timetable for laboratory preparation of QWA samples into thin sections ready for imaging

The light grey areas indicate machine time, while the white areas represent operator time
